# Finding the Perfect Match: Investigation of 1,2‐Diketone‐Based Materials for Use as Cathode Active Material in Rechargeable Magnesium Batteries

**DOI:** 10.1002/smll.74342

**Published:** 2026-07-01

**Authors:** Noushine Dorrani, Hendrik Koger, Ingo Krossing, Daniel B. Werz

**Affiliations:** ^1^ Institute of Organic Chemistry Albert‐Ludwigs‐Universität Freiburg Freiburg Germany; ^2^ Institute for Inorganic and Analytical Chemistry Albert‐Ludwigs‐Universität Freiburg Freiburg Germany

**Keywords:** cathode, charge density, chemical engineering, cyclic voltammetry, electrolyte, magnesium, materials science, organic synthesis, polymer, redox

## Abstract

Motivated by the high capacity and energy enabled by the two‐electron donation of earth‐abundant magnesium, we developed a novel, generally applicable screening method to evaluate 1,2‐diketone‐based redox units as n‐type cathode active materials (CAMs) for rechargeable magnesium batteries (RMBs). Preliminary DFT calculations (redox potentials, charge density mapping) and cyclic voltammetry in supporting and magnesium electrolyte solutions were used to screen exemplary diketone classes, eliminating unsuitable candidates and identifying phenanthrenequinone (PNQ) as the most promising redox unit for incorporation into a vinylic polymer. Non‐conjugated PNQ‐based vinylic polymers were successfully synthesized, characterized, and tested as CAMs at the coin‐cell level. Non‐optimized Mg‐PNQ cells delivered a starting capacity of up to 77 mAh g^−1^, two high‐voltage discharge plateaus at 2.2 and 1.7 V *vs*. Mg^2^^+^/Mg, and capacity retentions of 65% after 50 cycles and 50% after 100 cycles, typical for Mg‐quinone systems. A novel electrolyte, [Mg_2_(Ohfip)_3_][Al(Ohfip)_4_] in DME, further improved performance to 91 mAh g^−1^ with 72% retention after 50 cycles. These results demonstrate that the presented screening methodology efficiently identifies suitable CAM candidates within broad substance classes and is readily transferable to further materials.

## Graphical abstract

A novel and generally applicable early screening method for 1,2‐diketones via cyclic voltammetry and DFT computations identifies phenanthrenequinone as a suitable candidate as cathode active material for use in RMB. Its successful incorporation into a polymer enabled promising lithium and magnesium battery performance.

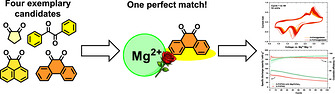

## 1 Introduction

Electrochemical energy storage methods, such as lithium‐ion batteries (LIB), play a key role in the energy transition [1, 2]. Their high round‐trip efficiency exceeding 90% makes them ideal candidates for a versatile alternative to pumped hydro storage [2, 3]. However, high production costs, safety concerns, and limited lithium resources motivate the development of more sustainable battery systems based on abundant metals such as aluminum, calcium, or magnesium [4, 5, 6]. The latter is particularly promising due to its diagonal relationship with lithium [7, 8, 9, 10], its high earth‐crust abundance of 2%, a low aqueous standard potential of –2.4 V, and a volumetric specific energy of 3861 mAh cm^−3^ arising from two‐electron redox processes [11, 12]. Although its gravimetric specific energy (2205 mAh g^−1^) is lower than lithium metal (3832 mAh g^−1^), it greatly exceeds that of the state‐of‐the‐art lithium/graphite system (372 mAh g^−1^) [12]. Like lithium, the low potential of magnesium *vs*. SHE requires non‐aqueous electrolyte systems [13, 14]. Initially, ether solutions of Grignard reagents RMgX were used as electrolytes, enabling magnesium plating/stripping without passivation layer formation [9, 15]. Their strongly basic and nucleophilic character, oxidative decomposition potential of ∼1.5 V *vs*. Mg^2^^+^/Mg, and low conductivity however, render them unsuitable for high‐potential cathodes or fast kinetics [16]. In the 1980s, adding aluminum‐centred Lewis acids to MgX_2_, RMgX, or MgR_2_ yielded improved electrolytes such as the trichloro‐bridged complex DCC or the all‐phenyl complex (APC) [9, 10, 17, 18, 19, 20, 21, 22]. Both exist in dynamic equilibrium containing nucleophilic Grignard species and/or corrosive AlCl_4_^−^ or Cl^−^, making them incompatible with most current collectors and cell casings [17, 23, 24]. In 2012, Mg[BH_4_]_2_ was introduced as an electrolyte salt for rechargeable magnesium batteries (RMB), with coulombic efficiencies (CE) up to 94% achieved using DME and Li[BH_4_] as an additive [25, 26, 27, 28]. Its electrochemical stability remains however limited, with an oxidation limit of only 1.7–2.3 V *vs*. Mg^2^^+^/Mg [27, 28]. Higher electrochemical stability of up to 4.0 V *vs*. Mg^2^^+^/Mg was achieved using weakly coordinating anions (WCAs), particularly highly fluorinated alkoxyborates and alkoxyaluminates developed by our group and and Strauss group [29, 30, 31, 32]. Notable examples include [Mg(dme)_3_][B(Ohfip)_4_]_2_ (Zhao‐Karger et al.), [Mg(dme)_3_][Al(Ohfip)_4_]_2_ (Herb et al.), and [Mg(thf)_7_][*pf*]_2_ (Lau et al.), with Ohfip = OC(H)(CF_3_)_2_ and [*pf*]^−^ = [Al(OC(CF_3_)_3_)_4_]^−^, all demonstrating good combinations of conductivity, stability, and CE in RMB [32, 33, 34, 35, 36, 37]. The aluminate [Mg(dme)_3_][Al(Ohfip)_4_]_2_ in DME is employed in this work [32, 38]. The challenge of suitable cathode active materials (CAM) nonetheless persists: NMC‐CAMs used in LIB contain critical elements (Co, Ni) and are incompatible with multivalent metals [39, 40, 41], while inorganic spinel‐ or olivine‐type materials suffer from structural instability during Mg^2^^+^ intercalation [42, 43, 44, 45]. Conversion‐type cathodes such as sulphur have been broadly investigated, but the polysulfide shuttle effect remains an unresolved challenge [45]. A promising alternative is organic materials, which are free from critical metals and exhibit more flexible properties than inorganic layered oxides, enabling faster kinetics and attracting growing interest as cathodes *vs*. lithium, aluminum, and magnesium [46, 47, 48]. The greatest challenge, however, remains the undesired solubility of the active material in the electrolyte solvent, leading to poor capacity retention and long‐term stability [46, 47]. One strategy to circumvent this is incorporating the redox‐active unit into a polymeric backbone, though this requires multi‐step synthesis [46, 47]. Two principal approaches exist: conjugated polymers, where redox‐active units are directly linked via covalent sp^2^–sp^2^ bonds, and non‐conjugated polymers, where they are separated by a redox‐inactive linker [47]. Conjugated polymers such as poly(aniline) (PANI) offer high conductivity but often produce heterogeneous voltages depending on chain length, and parasitic interactions between redox units can cause discrepancies between theoretical and practical capacity [49]. Non‐conjugated polymers with precisely defined, independent redox units can help prevent these issues. Organic CAMs are grouped into three categories by redox mechanism: p‐type (electron donors, oxidized), n‐type (electron acceptors, reduced), and b‐type materials, which can be both oxidized and reduced, enabling diverse energy storage mechanisms [47]. Bitenc et al. reported a functioning poly(1,4‐anthraquinone)/magnesium full‐cell with good stability in the Mg(TFSI)_2_/MgCl_2_ system, albeit at low voltage *vs*. Mg^2^^+^/Mg [50]. The 1,2‐diketone/enediol redox system is a promising n‐type alternative, as the reduced dianionic form is expected to form a five‐membered chelate complex with the metal cation, making it particularly suited to divalent cations such as magnesium or calcium [51, 52, 53, 54, 55, 56, 57]. In 2020, Pirnat et al. reported a conjugated poly(phenanthrenequinone)/reduced graphene oxide composite (PFQ/rGO) cathode in a Mg(TFSI)_2_/MgCl_2_‐based magnesium battery, achieving over 150 mAh g^−1^ over 450 cycles at 0.5 C [58]. However, after subtraction of the non‐negligible capacity contributions from rGO (80 mAh g^−1^) and carbon black (Printex XE2, 30 mAh g^−1^), the actual quinone contribution can be estimated to around 110 mAh g^−1^ [58, 59]. In the same year, Dong et al. reported a magnesium full cell using a pyrene‐tetraone‐based small molecule cathode (PTO) combined with a graphene oxide membrane to suppress dissolution [57]. In 2025, Yao et al. employed a similar PTO‐based cathode with a magnesiated NAFION‐ or SPEEK‐membrane separator, improving retention, though only demonstrated over five cycles [60]. Across all reported quinone‐based materials, only approximately half of the theoretical capacity was reached on the cell level, representing a recurrent challenge in RMBs. A literature overview on the capacities reached with quinone‐based cathodes in RMBs can be found in the Supporting Information.

## 2 Results and Discussion

### 2.1 Preliminary Investigations

**FIGURE 1:**
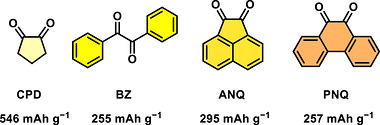
Investigated 1,2‐diketone‐based molecules along with their theoretical specific energy, calculated following Faraday´s law and a 2 e^–^ reduction [61].

In this work, exemplarily selected different 1,2‐diketone‐based materials were synthesized or purchased, and their suitability for use as CAM for RMBs was first investigated on the level of the isolated molecules. The four candidates, cyclopentadione (**CPD**), benzil (**BZ**), acenaphthoquinone (**ANQ**), and phenanthrenequinone (**PNQ**) are shown in Figure 1, along with their theoretical specific energy for 2 e^–^ processes.

**CPD**, **BZ**, **ANQ,** and **PNQ** represent a broad portfolio of different structural moieties that exhibit the common 1,2‐diketone motif. **CPD** is the only aliphatic candidate, exhibiting an extremely high theoretical capacity, due to its low molecular weight. **BZ** can be described as an aromatic 1,2‐diketone, which exhibits free rotation along the C1‐C2 axis. **ANQ** and **PNQ** are both *ortho*‐quinones with planar and rigid structures. They belong to the known quinone‐class of cathode active materials. Furthermore, all four candidates are easily accessible and present theoretical capacities in a competitive range. Hence, we were interested to differentiate between the suitability of these systems on the basis of a generally applicable, early screening method.

To find suitable redox units without having to go through the full polymer synthesis, battery fabrication, and electrochemical measurements, a ranking methodology was developed. It applies orienting investigations via quantum chemical calculations of the redox potentials, combined with cyclic voltammetry experiments of the monomers in solution, at first in an inert supporting electrolyte to verify the redox‐activity of the candidates, then in the weakly coordinating [Al(Ohfip)_4_]—RMB‐electrolyte to monitor the suitability of the candidate as CAM and select it for a study in RMB full cells. These preliminary investigations showed **PNQ** as the only promising candidate, which was further incorporated into non‐conjugated vinyl polymers.

#### 2.1.1 Cyclic Voltammetry (CV)

**FIGURE 2:**
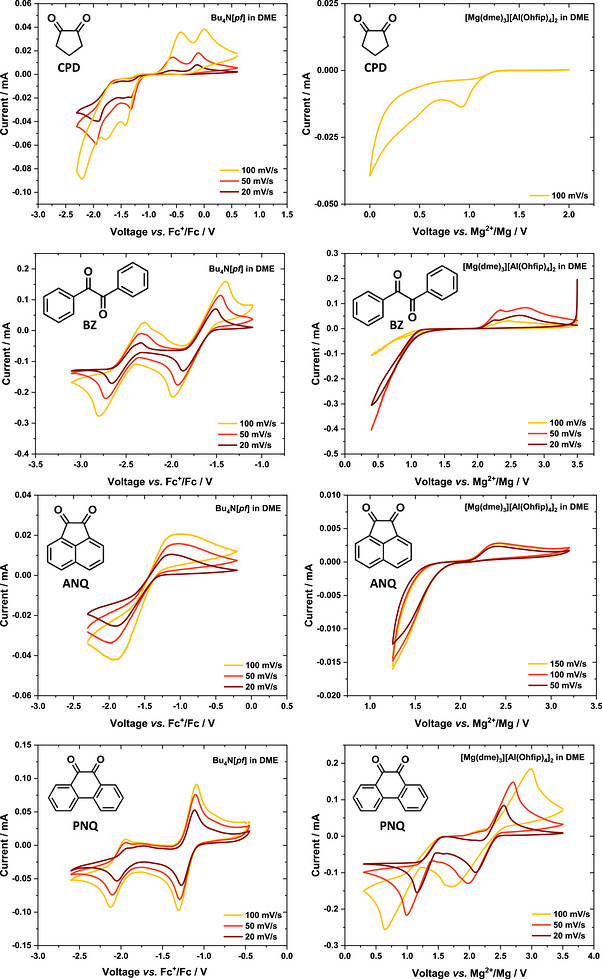
Cyclic voltammograms of the investigated 1,2‐diketones (10 mm) in two different electrolytes: The inert electrolyte with 100 mm Bu_4_N[*pf*] in DME (WE = Glassy Carbon 3 mm, CE = Pt, RE = Fc@Ag) and the RMB‐electrolyte with 300 mm [Mg(dme)_3_][Al(Ohfip)_4_]_2_ in DME (WE = Glassy Carbon 3 mm, CE = Mg, RE = Mg).

The cyclic voltammetry (CV) experiments were first conducted with 1 mm analyte in a solution of 100 mm Bu_4_N[*pf*] in DME as the electrolyte, using a glassy carbon (3 mm) working electrode or a platinum working electrode, a platinum counter electrode, and a reference compartment electrode equipped with a silver wire in an equimolar solution of 10 mm Fc and 10 mm Fc[*pf*] in the electrolyte. Thereafter, the CV experiments were conducted with 3 mm analyte in 300 mm [Mg(dme)_3_][Al(Ohfip)_4_]_2_ in DME, to mirror the conditions used in an RMB established within the group. These measurements were performed using a glassy carbon or platinum working electrode, and a magnesium ribbon as the respective counter and reference electrode. The resulting CVs are collected in Figure 2 and are discussed in the following. The cyclic voltammogram of cyclopentadione **CPD** *vs*. Fc^+^/Fc in an inert electrolyte system shows redox activity: two reduction waves at −1.31 and −1.95 V *vs*. Fc^+^/Fc, followed in the back scan by two oxidation waves at −0.44 and −0.05 V *vs*. Fc^+^/Fc (at 50 mV s^−1^). However, the hysteresis between reduction and oxidation, as well as the asymmetrical integration of reduction and oxidation processes, shows the redox reactions to be only quasi‐reversible. The measurements in the Mg‐electrolyte show one reduction at 0.92 V* vs*. Mg^2+^/Mg, followed by decomposition. This clearly excludes **CBD** from further testing in RMBs. By contrast, the benzil **BZ** CV shows two reversible redox processes at *E*_1/2_  =  −1.72 V and −2.55 V *vs*. Fc^+^/Fc. Our second test, the CV in the magnesium electrolyte, however, shows a reductive decomposition of the compound with a potential onset at 1.20 V *vs*. Mg^2+^/Mg, followed by a sluggish oxidation starting at about 2.00 V *vs*. Mg^2+^/Mg. It is likely that the decomposition products are oxidized. These characteristics render **BZ** also unsuitable for use in RMBs. The reductive decomposition might result from the forced conformational change along the C1−C2 axis, due to the formation of a chelate complex with magnesium, leading to an unfavored s‐*cis* conformation of the phenyl groups. The CV of acenaphthoquinone **ANQ** in the inert Bu_4_N[*pf*] electrolyte shows one quasi‐reversible redox process, with *E*_1/2_ at −1.54 V *vs*. Fc^+^/Fc. The second redox process was not observed. Potentially, this is induced by the Hückel anti‐aromaticity of the fully reduced form of **ANQ** with 12 π‐electrons. The CV in the Mg electrolyte shows redox activity with a reduction onset starting at 1.80 V *vs*. Mg^2+^/Mg and an oxidation maximum at 2.35 V *vs*. Mg^2+^/Mg. The absence of a second redox process and the irreversibility with Mg make **ANQ** unsuitable for RMBs. The CV of phenanthrenequinone **PNQ** in the inert electrolyte shows two redox processes, the first being reversible at *E*_1/2_  =  −1.19 V and the second occurring at *E*_1/2_  =  −2.04 V, both *vs*. Fc^+^/Fc. The second oxidation wave is more defined at higher scan rates, indicating a fast redox chemistry compared to diffusion. Now the CV in the Mg electrolyte also shows two redox processes: the first reversible at *E*_1/2_  =  2.32 V and the second quasi‐reversible at *E*_1/2_  =  1.33 V, both *vs*. Mg^2+^/Mg. The rather good (quasi‐) reversibility of the redox processes in the magnesium electrolyte, combined with the relatively high potential (at least for the first redox wave) *vs*. Mg^2+^/Mg, makes **PNQ** a promising candidate for further investigations.

#### 2.1.2 Calculations

##### 2.1.2.1 Electrostatic Plot and Density Charge

**FIGURE 3:**
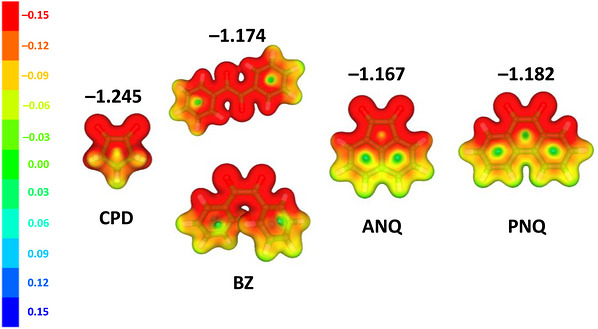
Calculated electrostatic potential plots in a.u. (Hartree · e^–^, isovalue of 0.025 e^–^ Bohr^ –^^3^) of the four investigated dianions of the 1,2‐diketone moieties **CPD**, **BZ** (two different conformations), **ANQ**, and **PNQ**, as well as calculated charges on the oxygen atoms of the dianions (charges in e, with e  =  1.6 · 10^–^^19^ C). The calculations (structure optimization, electrostatic potential plots, and QTAIM analysis) occurred at the RI‐BP86(D4)/def2‐TZVPP level of theory at 298 K.

In the following, we investigate the hypothesis of a causal relationship between the reversibility of a redox process in these 1,2‐diketones and the stabilization of the resulting charge upon reduction. Hence, the CV experiments were correlated with the calculated partial charges residing on the oxygen atoms of the fully reduced 1,2‐diketone moiety of the candidates. In addition, we expected that the more delocalized and hence stabilized the negative charges are, the smaller the partial charges on the oxygen atoms of the dianions will be. For visualization, the electrostatic potential plots of the dianions of the investigated molecules were calculated by RI‐BP86(D4)/def2‐TZVPP DFT calculations and are collected in Figure 3.

In all four compounds, the negative charges and the electron density are higher on the oxygen atoms, which are the redox‐active sites. However, as expected, the delocalization of the negative charges through resonance in the conjugated system of the aromatic candidates (**BZ**, **ANQ,** and **PNQ**) leads to regions where the electron density is lower, compared to **CPD**, which does not present aromatic resonance stabilization of the negative charges. Those qualitative results were confirmed by the QTAIM analysis (RI‐BP86(D4)/def2‐TZVPP) of the dianions, which yields robust partial charges that are rather insensitive to the method and basis set chosen. In agreement with this, the calculated partial charge of **CPD^2−^** of −1.245 is up to 0.078 higher than the partial charges of the two aromatic compounds. This potentially leads to stronger interactions with the magnesium cation and may account for the non‐reversibility of the reduction processes in the CV measurement in the magnesium electrolyte. All three aromatic compounds, **BZ**, **ANQ**, and **PNQ,** present comparable partial charges on the oxygen atoms, which agrees with the reversible redox processes observed in the Bu_4_N[*pf*] electrolyte *vs*. Fc^+^/Fc.

##### 2.1.2.2 Redox‐Potentials

Before calculating the redox potentials of the different candidates, a benchmarking study on **PNQ** was performed with different solvation models in DME (*ε*_r_ = 7.2) [62, 63]: CPCM from Orca (version 8.3.), COSMO from TurboMole, and COSMO‐RS with COSMOtherm. The structure optimizations and calculations were performed at the RI‐BP86(D4)/def2‐TZVPP level of theory, at 298 K. The redox potentials *vs*. Fc^+^/Fc were calculated as described in the supplementary information (SI) in Section S5. These calculated potentials were compared with the CV experiment in the standard electrolyte *vs*. Fc^+^/Fc. To obtain the calculated results *vs*. Fc^+^/Fc, the ferrocene assumption and the redox potential of ferrocene in CH_2_Cl_2_ (*E*_1/2 _ =  700 mV *vs*. SHE) [64] were used, since CH_2_Cl_2_ has a permittivity close to DME (*ε*_25°C_  =  8.9 (CH_2_Cl_2_); 7.2 (DME)) [12, 62]. The redox potentials obtained are then compared with the experimental values in Table 1.

**TABLE 1:** Calculated redox potential in V *vs*. Fc^+^/Fc of **PNQ** with different solvation models at 298 K and using the RI‐BP86(D4)/def2‐TZVPP level of theory.

Reduction	*E*_CPCM_		*E*_COSMO_	*E*_COSMO‐RS_	*E*_MEASURED_
PNQ/PNQ^·−^		0.99	0.97	−1.23	−1.19
PNQ^·−^/PNQ^2−^		−2.01	−2.03	−2.08	−2.04

Table 1 shows very similar results for CPCM and COSMO, which were expected, since both models are based on the conductor‐like polarizable continuum model [65, 66]. Both models were unable to describe the first reduction process in a sufficient way and predicted a redox potential more than 2 V higher than the experimental value. However, the calculated potential for the second reduction is acceptable, with less than 100 mV deviation from the experimental values. By contrast, COSMO‐RS, used with the COSMOtherm interface, yielded potential values for both redox processes with less than 40 mV difference from the experiment, well within the error interval of the CV measurement. Therefore, the combination of COSMO‐RS with COSMOtherm at the RI‐BP86(D4)/def2‐TZVPP level of theory is a suitable method for predicting redox potentials for organic 1,2‐diketone CAMs. This method was further applied to the other investigated 1,2‐diketones, except for **CPD**, as the poor reversibility of its CV makes it difficult to reliably assign the observed reduction waves to the corresponding theoretical reductions. The calculated redox potentials for the reductions of the three investigated compounds with reversible CV are compared with the measured CV values and collected in Table 2.

**TABLE 2:** Calculated redox potentials of the investigated candidates, compared with the experimental values. The calculations simulate a temperature of 298 K in DME solvent at the RI‐BP86(D4)/def2‐TZVPP level of theory using the Cosmo‐rs code.

	*E*_measured_ / V *vs*. Fc^+^/Fc	*E*_calculated_ / V *vs*. Fc^+^/Fc
PNQ/PNQ^·−^	−1.19	−1.23
PNQ^·−^/PNQ^2−^	−2.04	−2.08
ANQ/ANQ^·−^	−1.54	−1.63
ANQ^·−^/ANQ^2−^	Not observed	−3.67
BZ/BZ^·−^	−1.72	−1.78
BZ^·−^/BZ^2−^	−2.55	−2.68

This method and this basis set, combined with the solvation model, allowed the calculation of the redox potentials of both electronation processes for most of the investigated 1,2‐diketones (**PNQ**, **ANQ**, and **BZ**) within 40—130 mV. Interestingly, the second reduction of acenaphthoquinone, which was not observed in the CV, was calculated to occur at the very low potential of −3.67 V *vs*. Fc^+^/Fc. This supports the hypothesis of the second reduction being thermodynamically unfavorable, because of the formation of a 12 π Hückel anti‐aromatic compound. CV combined with quantum chemical calculations suggests that **PNQ** is the most promising candidate that will be further investigated for use as a non‐conjugated vinylic CAM‐polymer in RMBs.

### 2.2 Phenanthrenequinone Modifications

#### 2.2.1 Substitution Pattern for Vinylic Polymers

**FIGURE 4:**
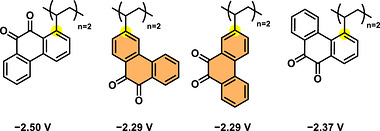
The four different possible symmetrical dimers and their calculated secondary redox‐potentials *vs*. Fc^+^/Fc, calculated at the RI‐BP86(D4)/def2‐TZVPP level of theory, CPCM solvation model for DME.

The question of the substitution pattern for the vinylic polymers remains. Four different derivatives are possible, depending on the position of the vinylic double‐bond, shown in Figure 4. To find the substitution pattern with the least negative influence on the electronic properties, the redox potential of the last reduction of the corresponding symmetrical dimers was calculated *vs*. Fc^+^/Fc using Orca (CPCM), since it yielded good results for the fully reduced second redox wave with the least amount of computational work.

The calculated redox potentials showed the *meta‐* and *para*‐substitution as being less thermodynamically detrimental for the redox potential, with a potential of −2.29 V *vs*. Fc^+^/Fc. It is, in fact, remarkable that the *ortho*‐dimer exhibits a potential of −2.50 V *vs*. Fc^+^/Fc, representing a loss of 210 mV compared with the most advantageous dimers. This highlights the need to investigate the ideal substitution pattern *a priori*.

#### 2.2.2 Materials Synthesis

**SCHEME 1:**
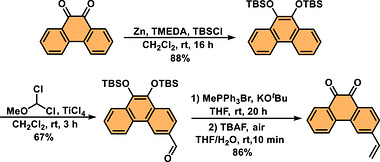
Synthetic route to the desired monomer via protection, formylation, olefination and deprotection. TMEDA = Me_2_NCH_2_CH_2_NMe_2_; TBS = Si*^t^* BuMe_2_; TBAF = [N(*^n^* Bu)_4_]F.

The monomer synthesis was performed via the introduction of a vinylic double bond to the commercially available **PNQ** in *meta‐* or *para*‐position. This was performed using straightforward procedures. Scheme 1 describes the synthesis of the *para*‐vinyl‐monomer, and the SI, Section 2, discloses full details. The desired monomer was prepared in three steps, starting with the in situ reduction of phenanthrenequinone to the dianionic species, followed by silyl‐protection. The use of the bulky TBS protecting groups allowed induction of a regioselective formylation toward the desired *para*‐product, compared to the methoxy‐protected analog, described by Speck, which yielded a 1:3 mixture of *ortho*‐ and *para*‐formylated products [67]. A Wittig reaction followed by deprotection under air yielded the desired monomer.

**SCHEME 2:**
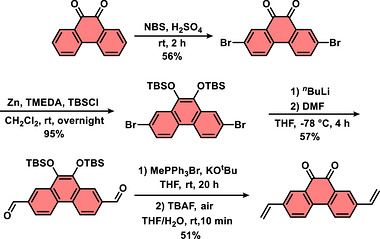
Synthetic route to the cross‐linker via bromination, protection, formylation, olefination, and deprotection. NBS = *N*‐bromosuccinimide.

To obtain an even less soluble polymer, a potential strategy is the use of a cross‐linker, containing two terminal vinylic double bonds (*e.g*., divinylbenzene), to form a polymer network instead of linear chains, after polymerization. However, this leads to the presence of redox‐inactive mass in the material, decreasing the specific energy of the CAM. To circumvent this issue, a phenanthrenequinone‐based cross‐linker was synthesized as shown in Scheme 2.

**SCHEME 3:**
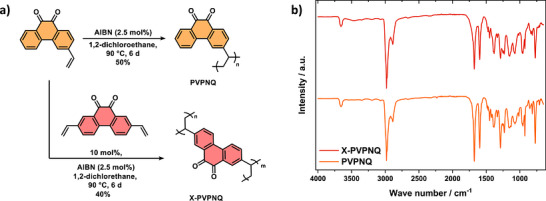
(a) Free radical polymerization of monomer to the non‐conjugated polymer **PVPNQ**, as well as the crosslinked polymer **X‐PVPNQ**. (b) ATR‐IR spectra of **PVPNQ** and **X‐PVPNQ** demonstrating the similarity and hence purity of both materials.

For synthetic reasons, both of the vinylic double bonds were incorporated in the *meta*‐position, which does not have a detrimental influence on the redox‐potential of the polymers, as shown by the calculations. The synthesis of the cross‐linker was achieved in a slightly different way than the synthesis of the main monomer, due to the impossibility of an Olah *bis*‐formylation. Phenanthrenequinone was brominated twice in the *meta*‐position. Then, the TBS protection was performed analogously to the synthesis of the main monomer. The incorporation of the carbaldehydes occurred via lithium‐bromine exchange and reaction with dimethylformamide. This was followed by Wittig reaction and deprotection under air to yield the desired cross‐linker. The polymerization reactions occurred via free radical polymerization, as shown in Scheme 3. The polymers were yielded as poorly soluble orange powders. The linear polymer **PVPNQ** could be characterized via ^1^H‐NMR, as well as size exclusion chromatography (SEC) and MALDI‐TOF. SEC indicates a *M*_w_ of 1.4·10^3^ Da, corresponding to six repeating units. However, those results might be underestimated because of the great insolubility of the system, even in chlorinated solvents, leading to only smaller oligomers dissolving and being measured.

A MALDI‐TOF mass spectrum shows the expected peak distance of *m/z* 234, corresponding to the mass of the monomer, between the detected species, with a highest *m/z* of 2.6·10^3^ (ca. 11 repeating units). The cross‐linked polymer **X‐PVPNQ** was completely insoluble and could only be characterized via IR spectroscopy, as well as cyclic voltammetry of the electrode‐processed polymer. ATR‐IR bands of **PVPNQ** and **X‐PVPNQ** bulk materials, as well as cyclic voltammograms of **PVPNQ** and **X‐PVPNQ** electrodes, are identical, indicating the successful synthesis of a cross‐linked polymer with the same electrochemical behavior, which only differs from the linear compound in solubility. The corresponding figures can be found in the Supporting Information.

#### 2.2.3 Electrochemical Characterization

To emphasize the need to incorporate the redox‐active unit in a polymeric backbone and to illustrate the consequences of a shuttle effect, the isolated small molecule (**PNQ**) was used as a model system for active material with high solubility. However, all battery quadruplicates stopped showing electrochemical activity during the first cycle, as shown in Figure S16. The post mortem analysis (Figures S17–S19) via IR study of the surface of the anode as well as of the cathode showed the consequence of the immediate solubilization of the active material in the electrolyte, leading to its diffusion to the anode and its deposition on the magnesium surface, thus emphasizing the need to incorporate the redox‐active unit into a polymeric backbone and attain immobilization on the cathode.

**FIGURE 5:**
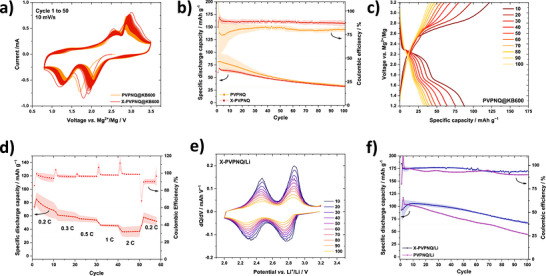
Electrochemical investigation of **PVPNQ**/Mg‐coin cells. WE: **(X‐)PVPNQ**@KB600 (90wt%)/PVdF (10 wt.%), CE: Mg‐disc, 3 x GF/A separators soaked with 60 µl of 300 mm [Mg(dme)_3_][Al(Ohfip)_4_]_2_ in DME. (a) Cyclic voltammetry at 10 mV/s on a range 0.8–3.5 V *vs*. Mg^2+^/Mg. (b) Charge/Discharge curves at 0.5 C between 1.2–3.2 V *vs*. Mg^2+^/Mg. (c) Constant current cycling test at 0.5 C between 1.2–3.2 V *vs*. Mg^2+^/Mg. (d) Rate capability test of **PVPNQ**/Mg cells between 1.2–3.2 V *vs*. Mg^2+^/Mg. (e) Electrochemical investigation of **PVPNQ**/Li half cells. WE: **(X‐)PVPNQ**@KB600 (90wt.%)/PVdF (10 wt.%), CE: Li‐disc, 3 x GF/A separators soaked with 60 µL of 300 mm LiPF_6_ in DME. dQ/dV *vs*. Voltage plots on a range 2.0–3.2 V *vs*. Li^+^/Li at 0.5 C. (f) Constant current cycling test at 0.5 C between 2.0–3.2 V *vs*. Li^+^/Li.

Hence, the synthesized polymers **PVPNQ** and **X‐PVPNQ** were processed into composite electrodes containing 45 wt.% active material, 45 wt.% Ketjenblack600D and 10 wt.% PVdF binder. The detailed electrode preparation is described in Section S1. 2032 Coin cells were fabricated and were characterized via cyclic voltammetry and constant current cycling tests. Furthermore, lithium half‐cells were fabricated to illustrate the performance of the cathode material with a well‐investigated anode system. For the sake of reproducibility, the cathodes used in the magnesium and lithium coin cells stemmed from the exact same batch. The obtained results are depicted in Figure 5. Figure 5a shows the cyclic voltammogram of **PVPNQ**‐ and **X‐PVPNQ**‐based coin cells in a potential range of 0.80–3.50 V *vs*. Mg^2+^/Mg at a scan rate of 10 mV s^−1^. As expected, two reduction waves are present, with peak maxima at 1.3 and 2.0 *vs*. Mg^2+^/Mg, followed by re‐oxidations at 2.5 and 2.8 *vs*. Mg^2+^/Mg for both materials. The CVs show a first cycle different from the rest, probably due to bulk reorganization of the polymers. The following 49 cycles show very similar behavior, indicating no major phase change or need for formation. Figure 5c shows the reversible charge and discharge curves of a **PVPNQ**‐based cell. As expected from the CV, two distinct discharge plateaus are evident from Figure 5c, with onsets at 2.3 and 1.7 V *vs*. Mg^2+^/Mg, whereas the charge occurs in one plateau, with an onset at 2.5 V *vs*. Mg^2+^/Mg. However, Figure 5c also reveals a fast capacity decay, apparently due to the solubility of the linear polymer in the electrolyte. In fact, the charge and discharge curves of cycle 100 occur in the form of a straight line, instead of a plateau. This indicates the complete loss of active material, and the remaining residual capacity presumably results from the capacitive behavior of the conductive carbon.

Figure 5b depicts the discharge capacity as well as the Coulombic efficiency against the number of cycles for both **PVPNQ** and **X‐PVPNQ**‐based systems in constant current cycling at a rate of 0.5 C (=  0.07 mA). **PVPNQ**‐based cathodes show a starting discharge capacity of 81 mAh g^−1^, which rapidly decreases, reaching 48 mAh g^−1^ after 50 cycles (60% retention), then 33 mAh g^−1^ after 100 cycles (40% retention). **X‐PVPNQ**‐based cathodes started with a lower discharge capacity of 68 mAh g^−1^, probably due to the difficult access to the cross‐linked active unit. The cells reached 45 mAh g^−1^ after 50 cycles (66% retention), then 32 mAh g^−1^ after 100 cycles (47% retention). Interestingly, **X‐PVPNQ** performed not only better in capacity retention over time, but also in reproducibility and Coulombic efficiency (average over 100 cycles: 89% *vs*. 82%). Figure 5d shows a rate capability experiment starting at 0.2 C, then 0.3 C, 0.5 C, 1 C and up until 2 C. The relatively low C‐rates compared with Li‐based systems are common for use in magnesium batteries, due to the high charge density of the magnesium cation, which hinders a fast‐stripping process. The cycling at 0.2 C after 50 cycles at higher C‐rates showed recovery of the capacity. Figure 5e shows the dQ/dV plot *vs*. voltage of the lithium half cells. As expected, it includes two redox‐processes. Interestingly, they exhibit way better reversibility compared to the magnesium system, as highlighted by the peak‐to‐peak distance of both redox‐processes. This may be attributed to the very high charge density of magnesium cations, which leads to strong interactions with the cathode material and explains the need for higher oxidative potentials to achieve *de‐magnesiation* upon charge. Figure 5f shows a constant current cycling test at 0.5 C of lithium half‐cells. Here, the starting capacity of cross‐linked material **X‐PVPNQ** in the very first cycle is 92 mAh g^−1^. During a formation phase of 10 cycles, it increases until 105 mAh g^−1^. This is over 30 mAh g^−1^ higher than the same cathode in the tested magnesium system. The capacity retention of the lithium‐half cells, as well as the coulombic efficiency, is also better, with 63% retention after 100 cycles, and an average coulombic efficiency of 95%. As expected, **PVPNQ**‐based half‐cells started with a capacity of 94 mAh g^−1^, followed by a shorter formation phase of 3 cycles, reaching 103 mAh g^−1^, but with poor capacity retention of 42% after 100 cycles, which is coherent with the above‐mentioned solubility issues. The better longevity of the lithium half‐cells compared to the magnesium cells contradicts the hypothesis that the poor capacity retention of the Mg cells is only due to intrinsic properties of the organic material. One complementary explanation could be the strong interactions between the negatively charged cathode material and the divalent magnesium cations. In fact, compared to the weakly coordinating hfip‐aluminate anions, the anionic quinone polymers are better ligands for the DME solvated magnesium cations than both counterion and solvent. This strong binding could presumably even lead to mass transport of the magnesium/quinone complex from the cathode into the electrolyte, leading to the observed poorer capacity retention.

**FIGURE 6:**
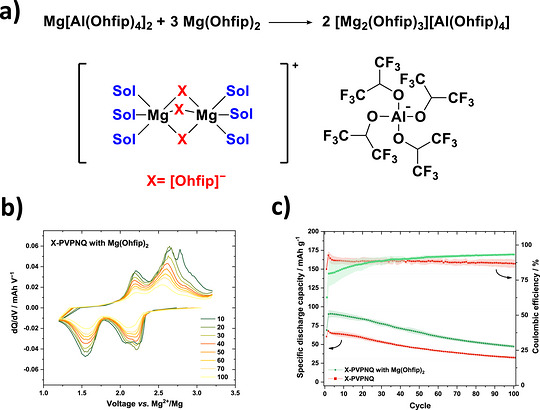
Novel electrolyte based on Mg(Ohfip)_2_ and Mg[Al(Ohfip)_4_]_2_. (a) Proposed reaction equation. (b) Electrochemical investigation of **PVPNQ**/Mg‐coin cells. WE: **(X‐)PVPNQ**@KB600 (90wt.%)/PVdF (10 wt.%), CE: Mg‐disc, 3 x GF/A separators soaked with 60 µL of 300 mm [Mg_2_(Ohfip)_3_][Al(Ohfip)_4_] in DME. dQ/dV plots *vs*. voltage at 0.5 C between 1.2–3.2 V *vs*. Mg^2+^/Mg. (c) Constant current cycling test at 0.5 C between 1.2–3.2 V *vs*. Mg^2+^/Mg.

This hypothesis motivated the need to provide magnesium with better ligands, without it leading to too much coordination, which would prevent the stripping process. Our idea was to use a stoichiometric 3:1 ratio of the magnesium hfip‐alcoholate Mg(Ohfip)_2_ and the magnesium hfip‐aluminate Mg[Al(Ohfip)_4_]_2_ to achieve a novel type of magnesium electrolyte, based on a formally singly positively charged magnesium complex, as shown in Figure 6a. Characterization via NMR spectroscopy of this novel magnesium system can be found in the supporting information. Magnesium coin cells were fabricated analogously to the above‐mentioned batteries, and tested via constant current cycling test, with **X‐PVPNQ** cathodes from the very same batch. The electrochemical results are included in Figure 6b. The resulting dQ/dV plot *vs*. voltage shows a similar redox‐activity than the CV (Figure 5a), but with better reversibility, as highlighted by the oxidation maxima at 2.2 and 2.6 V compared to 2.5 and 2.9 V in the standard system. Moreover, the cycling performances are also more promising with **X‐PVPNQ**/Mg cells showing a higher starting capacity of 91 mAh g^−1^ and achieving 50% retention after 100 cycles. However, the average coulombic efficiency is slightly lower with this novel electrolyte (82% over 100 cycles), but with a clear increase over the first 30 cycles, then reaching a plateau at 90% coulombic efficiency.

The use of this novel electrolyte could help achieve magnesium battery performances which were closer to the lithium half‐cells by reducing the charge density on the magnesium cation. However, the capacity retention as well as reversibility still leave room for improvement.

## 3 Conclusion

In summary, we report a screening of various 1,2‐diketone‐based redox‐active molecular units as potential candidates for use as cathode active materials in rechargeable magnesium batteries, relying on a resource‐friendly methodology consisting of DFT calculations combined with cyclic voltammetry experiments of the isolated molecules. Those investigations allowed to declare **CPD**, **BZ**, and **ANQ** as unsuitable. It is worth noting that the reason for the unsuitability of **ANQ** is the anti‐aromatic 12 π character of its dianion, which, according to the benchmark DFT calculations, would require a very negative potential of –3.67 V *vs*. Fc^+^/Fc to get doubly reduced. By contrast, **PNQ** emerged from these investigations as a promising redox‐active unit, due to reversible redox processes, favorable delocalization of the resulting charges over the aromatic system, combined with a high potential enabled by chelation of magnesium. Hence, **PNQ**‐based non‐conjugated polymers (linear **PVPNQ**, and cross‐linked **X‐PVPNQ**) were successfully synthesized, fully characterized, and further investigated at the coin‐cell level in magnesium full‐cells with the hfip‐aluminate‐based electrolyte operating in DME. The non‐optimized batteries showed a promising starting capacity of up to 77 mAh g^–^^1^, combined with two high‐voltage discharge plateaus at 2.2 and 1.7 V *vs*. Mg^2+^/Mg combined with a capacity retention of up to 65% after 50 cycles. Insights gained by a comparative study of lithium half‐cells yielded a better understanding of the cathode materials and highlighted the responsibility of the high charge density of magnesium in the capacity loss over time. This motivated the need to develop a novel electrolyte system [Mg_2_(Ohfip)_3_][Al(Ohfip)_4_] in DME, which yielded a promising starting capacity of 91 mAh g^−1^ combined with a capacity retention of 72% after 50 cycles. Full battery characterization of an optimized system will be the subject of future investigations. The developed workflow and methodology were proven efficient to highlight suitable candidates among a broad substance class, and can be easily applied to further materials with very few adjustments, for instance by investigating the delocalization of the positive charge in p‐type materials.

## Conflicts of Interest

The authors declare no conflicts of interest.

## Supporting information




**Supporting File**: smll74342‐sup‐0001‐SuppMat.docx.

## Data Availability

The data supporting this article have been included as part of the supplementary information (SI).
