# Supplementary material for: Finding the Perfect Match: Investigation of 1,2‐Diketone‐Based Materials for Use as Cathode Active Material in Rechargeable Magnesium Batteries
Source: Small. 2026 Jul 1;22(46):e74342. doi: 10.1002/smll.74342 (PMC13484997; doi:10.1002/smll.74342)
Supplement: Supplementary file 1 [file SMLL-22-e74342-s001.docx]

Finding the Perfect Match: Investigation of 1,2‑Diketone-Based Materials for Use as Cathode Active Material in Rechargeable Magnesium Batteries

Noushine Dorrani, Hendrik Koger, Ingo Krossing* and Daniel B. Werz*

**Supporting Information**

Contents

[1.	Materials and methods	2](#_Toc230854835)

[2.	Literature overview	4](#_Toc230854836)

[3.	Synthetic procedures	4](#_Toc230854837)

[4.	NMR spectra	9](#_Toc230854838)

[5.	NMR Characterization of [Mg_2_(Ohfip)_3_][Al(Ohfip)_4_] in DME	14](#_Toc230854839)

[6.	Polymer characterisation	15](#_Toc230854840)

[7.	Electrochemical measurements	19](#_Toc230854841)

[8.	Quantum chemical calculations	23](#_Toc230854842)

[9.	Cartesian coordinates of optimized structures	25](#_Toc230854843)

[10.	X-Ray data	32](#_Toc230854844)

# 1. Materials and methods

## Chemicals

Magnesium ribbon (99.5 %, Sigma Aldrich) and magnesium foil (99.95 %, thickness: 0.1 mm, *d* = 15 mm for coin cells and Changsha Rich Nonferrous Metals Co.,Ltd) were polished with glass slides before usage. 1,2‑Dimethoxyethane (for HPLC, 99.9 %, Sigma‑Aldrich) was dried over technical grade LiAlH_4_ and distilled before usage and stored over 3 Å molecular sieve in an argon filled Schlenk vessel. Chemicals and reagents were purchased from Acros-Organics, Alfa-Aesar, Chempur or Sigma Aldrich. Unless otherwise noted, all substrates, reagents and solvents were taken from commercial sources and used without further purification. Solvents denoted as “anhydrous” in the following were obtained from an *MB-SPS-800* solvent purification system by MBRAUN and stored over activated molecular sieves (3 Å). *n*-Pentane and ethyl acetate (EtOAc) were purchased in technical grade and distilled prior to use.

## Nuclear Magnetic Resonance Spectroscopy

All NMR spectra were recorded either using Bruker *DRX 400 MHz*, Bruker *Avance VIII HD 300 MHz* spectrometers. Measurement conditions (NMR core, resonance frequency, solvent) are given with the respective spectrum. Evaluation followed first order using Mestrenova software. Report of NMR signals is given using the following order: chemical shift in ppm (multiplicity (s = singlet, d = doublet, t = triplet, q = quartet, quint. = quintet, sept. = septet, m = multiplet and possible combinations thereof), coupling constant(s) *^n^J*_A, B_ in Hz, integration (for ^1^H) and specification of the respective core, the signal originated from). Chemical shifts are given in *parts per million* (ppm, δ‑scale) and are referenced using literature (Fulmer *et al.*) known shifts in ^1^H‑NMR of the respective solvents.^[1]^ Calibration of heteronuclear spectra was performed according to corresponding ^1^H NMR spectra.

## PVPNQ Composite Electrodes

Pre‑dried PVPNQ or X-PVPNQ (45 wt‑%) and conductive carbon black (Ketjenblack®600D) (45 wt‑%) were mortared together, solubilized in CH_2_Cl_2_ (5 ml) and sonicated for 10 min. The suspension was then filtered and washed with CH_2_Cl_2_ before drying. The obtained composite PVPNQ@KB600 (90 wt%), as well as PVdF binder (Sigma‑Aldrich)(10 wt‑%) were mixed and dissolved in NMP. The slurry was homogenized using a Thinky *ARM‑310* planetary mixer at 2000 rpm for 30 min, then cast onto a KOH-etched aluminum foil (1235 aluminum foil, H18 hard state, 20 µm, Gelon LIB Group, etched for 1 min in an aqueous KOH solution) using a blade‑coating technique. The resulting coated sheets were dried in a vacuum drying oven at 60 °C for 24 h. Coin cell electrodes were punched using an EL‑Cell *EL‑Cut* manual punching machine (15 mm diameter) and dried *in vacuo* (10^−3^ mbar, 120 °C, 24 h). Active material mass loading (average over 4 electrodes) of 0.5830 ± 0.0247 mg was achieved.

## Electrochemical Measurements

Battery cycling was performed at 25 °C using a Landt instruments *CT3002AU‑5V‑50mA* battery tester installed in an actively temperature-controlled housing (Binder *KB115 Cooling Incubator*). Cycling conditions (current, cut‑off voltages) are given below for the corresponding experiments. Landt instruments *LANDProc V7.4* was used for setting of cycling programs, Landt instruments *LANDMon V7.4* was used for surveillance, control and data recording, Landt instruments *LANDdt V7.4* was used for data processing and OriginLab *OriginPro 2025* for plotting and graphics. For each cell group, two identical coin cells were fabricated, using Mg foils (100 µm, *d* = 15 mm) scraped with the edge of glass slides, three layers of GF/A separators soaked with 60 µL of a 0.3 m solution of [Mg(dme)_3_][Al(O*hfip*)_4_]_2_ in DME, a PVPNQ electrode. The coin cells were pressed with 1000 psi. As a standard procedure, OCV was recorded for 5 min before cycling. All cyclic voltammetry measurements were performed in an argon filled glovebox, using BioLogic *VMP‑3* potentiostat and BioLogic *EC‑Lab V11.50* software. Between each voltage sweep rate change, OCV was recorded for 30 s. Evaluation was performed using BioLogic *EC‑Lab V11.50* software and OriginLab *OriginPro 2025* for plotting and graphics. The cycling data shows the average data for two identical coin cells.

# 2. Literature overview

Table S1: Literature overview on quinone-based cathodes for use in magnesium batteries published in the past years.*

| Material | Theoretical capacity  /mAh g^–1^ | Exp. capacity  / mAh g^–1^ (%) | Electrolyte |
| --- | --- | --- | --- |
| P14AQ^[2]^ | 250 | <150 (<60%)* | MgCl_2_-AlCl_3_ |
| P14AQ^[2]^ | 250 | 150 (60%)* | Mg(TFSI)_2_/MgCl_2_ |
| PFQ/rGO^[3]^ | 250 | 150 (60%)* | Mg(TFSI)_2_/MgCl_2_ |
| PAQS^[4]^ | 225 | 105 (45%) | MHCC |
| PTO^[5]^ | 410 | 200 (48%) | MMC/G4 |
| PAAQ^[6]^ | 300 | 140 (45%) | Mg(TFSI)_2_ |
| PPTS^[7]^ | 300 | 150 (50%) | Mg(B(Ohfip)_4_)_2_ |

*Reported capacities without subtraction of the relevant carbon black contribution of ca. 30 mAh g^–1^ and the even more relevant rGO contribution in the PFQ/rGO system.

Analysis of Table S1 clearly signals that the after consideration of the capacity contributions of carbon black and rGO, all resulting corrected exp. capacities only reach around 50 % of the theoretical expectation value. This is content to intense investigation in our group currently and will be subject to a separate publication.

# 3. Synthetic procedures

1,2-Cyclopentanedione, 2,7-Dibromophenanthrene-9,10-dione and ((2,7-dibromophenanthrene-9,10-diyl)bis(oxy))bis(*tert*-butyldimethylsilane) were synthesised following known procedures.^[8]^

## Synthesis of 9,10-Bis((*tert*-butyldimethylsilyl)oxy)phenanthrene

Phenanthrene-9,10-dione (3.00 g, 14.4 mmol, 1.0 eq.) was suspended in dry dichloromethane (350 mL) at room temperature. Zink powder (14.1 g, 216 mmol, 15 eq.) was added, followed by TMEDA (11.0 mL, 72.0 mmol, 5.0 eq.) and TBSCl (10.0 mL, 57.6 mmol, 4.0 eq.). The resulting suspension was stirred for 20 h at room temperature. The reaction mixture was filtered through a Celite pad and the solvent was removed under reduced pressure. The obtained crude solid was purified *via* column chromatography (SiO_2_, pentane, R_f_ = 0.8). The product (5.56 g, 12.7 mmol, 88%) was afforded as an off-white powder. **^1^H NMR** (400 MHz, CDCl_3_): δ = 8.62-8.59 (m, 2 H, Ar), 8.22-8.20 (m, 2 H, Ar), 7.59-5.52 (m, 4 H, Ar), 1.15 (s, 18 H, *^t^*Bu), 0.09 (s, 12 H, Me) ppm. **^13^C NMR** (100 MHz, CDCl_3_): δ = 137.5, 130.5, 127.8, 126.0, 124.9, 123.1, 122.5, 26.6, 25.7, 18.8, 0.10, −3.46, −3.54 ppm. **HRMS** (APCI+): *m/z* calcd. for C_26_H_39_O_2_Si_2_ 439.2483 [M+H]^+^, found 439.2483 [M+H]^+^. **IR** (ATR): *ṽ* [cm^–1^] = 2957, 2935, 2857, 1595, 1447, 1331, 1251, 1173, 1111, 1058, 1028, 920, 826, 779, 755, 725, 674. **m.p.**: 177.8 °C.

## Synthesis of 9,10-Bis((*tert*-butyldimethylsilyl)oxy)phenanthrene-3-carbaldehyde

A solution of 9,10-Bis((*tert*-butyldimethylsilyl)oxy)-phenanthrene (3.00 g, 6.83 mmol, 1.0 eq.) in dry CH_2_Cl_2_ (50 mL) was brought to 0 °C. TiCl_4_ (1.2 mL, 10.6 mmol, 1.5 eq.) was added dropwise, followed by dichloromethyl methyl ether (0.8 mL, 9.57 mmol, 1.4 eq.). The purple solution was warmed to room temperature and was stirred overnight. Water (30 mL) was added to quench the reaction. After extracting with CH_2_Cl_2_ (3 x 100 mL), the combined organic layers were washed with water and brine, dried over Na_2_SO_4_, filtered and evaporated under reduced pressure. The obtained crude solid was purified by column chromatography (SiO_2_, *n*-pentane/ethyl acetate 10:1, R_f_ = 0.3). The product (2.15 g, 4.60 mmol, 67%) was afforded as a colourless powder. **^1^H NMR** (400 MHz, CDCl_3_): δ = 10.24 (d, *J* = 0.6 Hz, 1 H, CHO), 9.10 (d, *J* = 1.3 Hz, 1 H, Ar), 8.73-8.68 (m, 1 H, Ar), 8.32 (dd, *J*_1_ = 8.5 Hz, *J*_2_ = 0.6 Hz, 1 H, Ar), 8.27-8.22 (m, 1 H, Ar), 8.05 (dd, *J*_1_ = 8.5 Hz, *J*_2_ = 1.7 Hz, 1 H, Ar), 7.66-7.62 (m, 2 H, Ar) 1.15 (s, 9 H, *^t^*Bu), 1.14 (s, 9 H, *^t^*Bu), 0.10 (s, 6 H, Me), 0.08 (s, 6 H, Me) ppm.**^13^C NMR** (100 MHz, CDCl_3_): δ = 192.3, 140.9, 137.5, 134.6, 132.9, 130.7, 128.1, 127.4, 127.0, 126.9, 125.9, 124.8, 123.9, 123.4, 122.6, 26.6, 18.9, 18.8, 0.08, −3.32, −3.39 ppm. **HRMS** (ESI+): *m/z* calcd. for C_27_H_39_O_3_Si_2_ 467.2359 [M+H]^+^, found 467.2438 [M+H]^+^. **IR** (ATR): *ṽ* [cm^–1^] = 2957, 2935, 2857, 1697, 1596, 1453, 1369, 1334, 1253, 1172, 1054, 922, 842, 781, 760. **m.p.**: 121.3 °C.

## Synthesis of 3-Vinylphenanthrene-9,10-dione

MePPh_3_Br (3.06 g, 8.57 mmol, 1.0 eq.) was slowly added to a solution of KO*^t^*Bu (1.06 g, 9.43 mmol, 1.1 eq.) in dry THF (40 mL) and the resulting orange suspension was stirred for 45 min at 15 °C, followed by the addition of 9,10-Bis((*tert*-butyldimethylsilyl)oxy)phenanthrene-3-carbaldehyde (4.00 g, 8.57 mmol, 1.0 eq.). The reaction mixture was stirred for 20 h at room temperature. Water (10 mL) was added, followed by TBAF (1 m solution in THF, 20 mL, 2.3 eq.) and the resulting brown suspension was stirred under air for 10 min at room temperature. Water (30 ml) was added and the mixture was extracted with CH_2_Cl_2_ (3 x 100 ml). The combined organic layers were combined, washed with water and brine, dried over Na_2_SO_4_, filtered and evaporated under reduced pressure. The obtained crude solid was purified by column chromatography (SiO_2_, CH_2_Cl_2_, R_f_ = 0.5). The product (1.73 g, 7.38 mmol, 86%) was obtained as an orange powder. **^1^H NMR** (400 MHz, CDCl_3_): δ = 8.20 (dd, *J*_1_ = 7.9 H, *J*_2_ = 1.5 Hz, 1 H, Ar), 8.16 (d, *J* = 8.0 Hz, 1 H, Ar), 8.05 (d, *J* = 8.1 Hz, 1 H, Ar), 7.98 (d, *J* = 1.8 Hz, 1 H, Ar), 7.73 (ddd, *J*_1_ = 7.5 Hz, *J*_2_ = 7.0 Hz, *J*_3_ = 1.8 Hz, 1 H, Ar), 7.51 (dd, *J*_1_ = 8.2, *J*_2_ = 1.8 Hz, 1 H, Ar), 7.48 (ddd, *J*_1_ = 7.8 Hz, *J*_2_ = 6.9 Hz, *J*_3_ = 1.2 Hz, 1 H, Ar), 6.83 (dd, *J*_trans_ = 17.5 Hz, *J*_cis_ = 10.8 Hz, 1 H, ArCH=CH_2_), 6.02 (d, *J*_trans_ = 17.6 Hz, 1 H, ArCH=CH_2_), 5.56 (d, *J*_cis_ = 10.8 Hz, 1 H, ArCH=CH_2_) ppm.**^13^C NMR** (100 MHz, CDCl_3_): 180.5, 179.8, 145.0, 136.3, 136.0, 135.9, 135.7, 131.3, 131.1, 130.6, 130.3, 129.7, 126.9, 123.9, 122.2, 118.7 ppm. **HRMS** (APCI+): *m/z* calcd. for C_16_H_11_O_2_ 235.0753 [M+H]^+^, found 235.0755 [M+H]^+^. **IR** (ATR): *ṽ* [cm^–1^] = 2957, 2935, 2857, 1670, 1590, 1477, 1446, 1396, 1289, 1235, 1172, 1037, 927, 850, 807, 774. **m.p.**: 168.9 °C (decomp.)

## Synthesis of 9,10-bis((*tert*-butyldimethylsilyl)oxy)phenanthrene-2,7-dicarbaldehyde

A solution of ((2,7-dibromophenanthrene-9,10-diyl)bis(oxy))bis(*tert*-butyldimethylsilane) (1.00 g, 1.68 mmol, 1.0 eq.) in dry THF (25 mL) was brought to −78 °C. A solution of *n*BuLi in hexanes (1 m, 2.6 mL, 4.19 mmol, 2.5 eq.) was added dropwise and the reaction mixture was stirred at −78 °C for 1 h. Dry DMF (0.7 mL, 8.38 mmol, 5.0 eq.) was added dropwise and the solution was stirred at rt for 4 h. Water (30 mL) was added to quench the reaction. After extracting with CH_2_Cl_2_ (3 x 100 mL), the combined organic layers were washed with water and brine, dried over Na_2_SO_4_, filtered and evaporated under reduced pressure. The obtained crude solid was purified by column chromatography (SiO_2_, pentane/CH_2_Cl_2_ 1:1, Rf = 0.3). The product (473 mg, 960 µmol, 57%) was afforded as bright yellow crystals. **^1^H NMR** (400 MHz, CDCl_3_): δ = 10.2 (s, 2 H, CHO), 8.79 (d, *J* = 1.8 Hz, 2 H, Ar), 8.76 (d, *J* = 8.6 Hz, 2 H, Ar), 8.08 (dd, *J*_1_ = 8.6 Hz, *J*_2_ = 1.8 Hz, 2 H, Ar), 1.19 (s, 18 H, *^t^*Bu), 0.12 (s, 12 H, Me) ppm. **^13^C NMR** (100 MHz, CDCl_3_): δ = 192.0, 138.8, 135.0, 131.7, 130.8, 128.2, 124.5, 123.4, 26.5, 18.9, −3.32 ppm. **HRMS** (ESI+): *m/z* calcd. for C_28_H_38_O_4_Si_2_ 495.2381 [M+H]^+^, found 295.2391 [M+H]^+^. **IR** (ATR): *ṽ* [cm^–1^] = 2938, 2720, 1701, 1575, 1428, 1380, 1313, 1232, 1144, 1103, 838, 805. **m.p.**: 114.6 °C (decomp.)

## Synthesis of 2,7-divinylphenanthrene-9,10-dione

MePPh_3_Br (974 mg, 2.79 mmol, 3.0 eq.) was slowly added to a cold solution of KO*^t^*Bu (412 mg, 3.64 mmol, 4.0 eq.) in dry THF (20 mL) and the resulting orange suspension was stirred for 45 min at 10°C, followed by the dropwise addition of 9,10-bis((*tert*-butyldimethyl-silyl)oxy)phenanthrene-2,7-dicarbaldehyde (450 mg, 910 µmol, 1.0 eq.). The reaction mixture was stirred for 20 h at room temperature. Water (10 mL) was added, followed by a TBAF solution (1 m in THF, 3.0 ml, 3.00 mmol, 3.0 eq.) and the mixture was stirred under air for 1 h. After extraction with CH_2_Cl_2_ (3 x 100 mL), the combined organic layers were washed with water and brine, dried over Na_2_SO_4_, filtered and evaporated under reduced pressure. The obtained crude solid was purified by column chromatography (SiO_2_, CH_2_Cl_2_, R_f_ = 0.5). The product (121 mg, 465 µmol, 51%) was obtained as a dark red powder. **^1^H NMR** (400 MHz, CDCl_3_): δ = 8.19 (d, *J* = 2.0 Hz, 2 H, Ar), 7.95 (d, *J* = 8.3 Hz, 2 H, Ar), 7.72 (dd, *J*_1_ = 8.4 Hz, *J*_2_ = 2.0 Hz, 2 H, Ar), 6.75 (dd, *J*_trans_ = 17.7 Hz, *J*_cis_ = 11.4 Hz, 2 H, ArCH=CH2), 5.92 (dd, *J*_trans_ = 17.7 Hz, *J*_gem_ = 0.9 Hz, 2 H, ArCH=CH2), 5.43 (dd, *J*_cis_ = 11.3 Hz, *J*_gem_ = 0.8 Hz, 2 H, ArCH=CH2) ppm. **^13^C NMR** (100 MHz, CDCl_3_): δ = 180.4, 138.9, 134.9, 134.9, 133.3, 131.1, 128.1, 124.4, 116.8 ppm. **HRMS** (APCI+): *m/z* calcd. for C_18_H_12_O_2_ 261.0910 [M+H]^+^, found 261.0907 [M+H]^+^. **IR** (ATR): ṽ [cm^–1^] = 2983, 2885, 1677, 1595, 1451, 1387, 1291, 1227, 1145, 1064, 960, 928, 848. **m.p.**: 147.7 °C.

## Synthesis of PVPNQ

AIBN (1.80 mg, 10.6 µmol, 2.5 mol%) was added to an Argon-purged, pre-heated solution of 3‑vinylphenanthrene-9,10-dione (100 mg, 427 µmol, 1.0 eq.) in dry 1,2-dichloroethane (0.5 ml). The mixture was heated at 90 °C for 6 d in a sealed vial. After cooling, the reaction mixture was poured in cold MeOH (10 mL) and the polymer precipitated. The solid was filtered, washed thoroughly with MeOH and dried in vacuo. The product (55 mg, 55%) was obtained as an insoluble dark orange powder. **^1^H NMR** (400 MHz, CDCl3): δ = 8.20-6.45 (br. m), 1.88-0.57 (br. m) ppm. **IR** (ATR): ṽ [cm^–1^] = 2983, 2885, 1677, 1595, 1451, 1387, 1291, 1227, 1145, 1064, 960, 928, 768. **m.p.:** 160 °C – 190 °C.

## Synthesis of X-PVPNQ

AIBN (3.60 mg, 21.2 µmol, 2.5 mol%) was added to an Argon-purged, pre-heated solution of 3‑vinylphenanthrene-9,10-dione (200 mg, 427 µmol, 1.0 eq.) and 2,7-divinylphenanthrene-9,10-dione (44.4 mg, 171 µmol, 20 mol%) in dry 1,2-dichloroethane (1.0 ml). The mixture was heated at 90 °C for 6 d in a sealed vial. After cooling, the reaction mixture was poured in cold MeOH (10 mL) and the polymer precipitated. The solid was filtered, washed thoroughly with MeOH, then CH_2_Cl_2_ and dried in vacuo. The product (220 mg, 97%) was obtained as an insoluble dark red powder. **IR** (ATR): ṽ [cm^–1^] = = 2983, 2885, 1677, 1595, 1451, 1387, 1291, 1227, 1145, 1064, 960, 928, 768. **m.p.:** 160 °C – 190 °C.

# 4. NMR spectra

Figure S1: ^1^H NMR (400 MHz, CDCl3) spectrum of 9,10-Bis((tert-butyldimethylsilyl)oxy)phenanthrene.

Figure S2: ^13^C NMR (100 MHz, CDCl3) spectrum of 9,10-Bis((tert-butyldimethylsilyl)oxy)phenanthrene.

Figure S3: ^1^H NMR (400 MHz, CDCl3) spectrum of 9,10-Bis((tert-butyldimethylsilyl)oxy)phenanthrene-3-carbaldehyde.

Figure S4: ^13^C NMR (100 MHz, CDCl3) spectrum of 9,10-Bis((tert-butyldimethylsilyl)oxy)phenanthrene-3-carbaldehyde.

Figure S5: ^1^H NMR (400 MHz, CDCl3) spectrum of 3-Vinylphenanthrene-9,10-dione.

Figure S6: ^13^C NMR (100 MHz, CDCl3) spectrum of 3-Vinylphenanthrene-9,10-dione.

Figure S7: ^1^H NMR (400 MHz, CDCl3) spectrum of 9,10-bis((tert-butyldimethylsilyl)oxy)phenanthrene-2,7-dicarbaldehyde.

Figure S8: ^13^C NMR (100 MHz, CDCl3) spectrum of 9,10-bis((tert-butyldimethylsilyl)oxy)phenanthrene-2,7-dicarbaldehyde.

Figure S9: ^1^H NMR (400 MHz, CDCl3) spectrum of 2,7-divinylphenanthrene-9,10-dione.

Figure S10: ^13^C NMR (100 MHz, CDCl3) spectrum of 2,7-divinylphenanthrene-9,10-dione.

# 5. NMR Characterization of [Mg_2_(Ohfip)_3_][Al(Ohfip)_4_] in DME

Figure S11: 1H NMR (400 MHz, DME) spectrum of [Mg_2_(Ohfip)_3_][Al(Ohfip)_4_].

Figure S12: 19F NMR (376 MHz) spectrum of [Mg_2_(Ohfip)_3_][Al(Ohfip)_4_].

# 6. Polymer characterisation

Figure S13: : Insolubility of the polymers **PVPNQ** and **X-PVPNQ** in CH_2_Cl_2_.

**X-PVPNQ**

**PVPNQ**

Figure S14: ^1^H NMR (400 MHz, CDCl_3_) spectrum of **PVPNQ**.

## Gel permeation chromatography: PVPNQ

## MALDI-TOF: PVPNQ

# 7. Electrochemical measurements

## Contribution of the conductive carbon to the capacity

KB600 (90 wt%) and PVdF binder (Sigma‑Aldrich)(10 wt‑%) were mixed and dissolved in NMP. The slurry was homogenized using a Thinky *ARM‑310* planetary mixer at 2000 rpm for 30 min, then cast onto a KOH-etched aluminium foil (1235 aluminium foil, H18 hard state, 20 µm, Gelon LIB Group, etched for 1 min in an aqueous KOH solution) using a blade‑coating technique. The resulting coated sheets were dried in a vacuum drying oven at 60 °C for 24 h. Coin cell electrodes were punched using an EL‑Cell *EL‑Cut* manual punching machine and dried *in vacuo* (10^−3^ mbar, 120 °C, 24 h). Those “dummy” cathodes were built in coin cells and tested *via* current cycling tests in the same manner than the PVPNQ-based cells. The specific discharge capacity against the number of cycles is depicted in Figure S13. The average discharge capacity due to conductive carbon over 500 cycles is 11.8 ± 0.5 mAh/g, and the average coulombic efficiency is 94 ± 2 %.

Figure S15: Constant current cycling test at 0.5 C on a range 1.2 - 3.2 V *vs*. Mg^2+^/Mg.
WE: KB600 (90wt%) and PVdF (10wt%), CE: Mg-disc,
3 x GF/A separators soaked with 60 µl of 300 mm [Mg(dme)_3_][Al(Ohfip)_4_]_2_ in DME.

## Comparative study of PNQ-based cathodes (small molecule)

Figure S16: Voltage *vs.* test time plot, illustrating no electrochemical activity, shown for one exemplary cell of the PNQ/Mg cell group.

To gather information about the surface processes inside the battery, all four cells from the PNQ-cell group were opened and the electrodes were investigated *via* ATR-IR spectroscopy. The obtained spectra were compared to the pristine materials (naked magnesium surface for the anode, and slurry before cycling for the cathode). The resulting spectra are depicted in Figure S17 and Figure S18. As shown in Figure S17, the composite electrode before the cycling contained the active material in the oxidized form, as shown by the two characteristic C=O bands at 1672 and 1591 cm^−1^. However, after the cycling, those two characteristic bands were absent in the IR spectrum, indicating the supposed leeching of the active material into the electrolyte. The novel bands at 1291, 1232, 1098 and 689 cm^−1^ are assigned to the electrolyte.^[9]^

**PNQ**

**Cathode after cycling**

**Cathode before cycling**

Figure S17: IR study of the cathode surface before and after cycling.

**Anode after cycling**

**Anode before cycling**

Background noise from Diamond ATR

Figure S18: IR study of the anode surface before and after cycling.

The hypothesis of a leaching can be confirmed by the IR study of the magnesium surface. As shown in Figure S18, the magnesium surface was completely passivated after the opening of the cell. Moreover, the IR spectrum of the magnesium surface compared with the IR spectra of the pristine magnesium surface showed new bands, indicating a deposition. By comparison of the IR spectra of the magnesium surface after the cycling and the measured spectra of PNQ as well as the calculated spectra of PNQ^2–^, an assignment of the new bands to the phenanthrenequinone dianion can be made, confirming the degradation of the organic material on the naked magnesium surface and thus the solubility.

Figure S19 Assignment of the novel bands on the magnesium surface after cycling.

# 8. Quantum chemical calculations

Unless stated otherwise, all quantum chemical calculations were implemented with *Orca* (version 5.0.4) or *Turbomole* (version 7.5.1).^[10]^ Structure optimization was performed with help of density functional theory (DFT)^[11]^, internal coordinates, resolution identity approximation RI^[12,13]^, D4‑dispersion correction^[14]^ and a fine grid size (grid size = m5). Calculations were carried out at the RI-BP86^[15]^(D4)/def2-TZVPP^[13,16]^ level of theory.

For each calculated species, standard enthalpy *H*^0^ at 298.15 K was calculated from electronic self-consistent field (SCF)^[17]^ energy *E*_SCF_ and free energy *E*_vrt_ using Equation (1).^[17,18]^

|  | $\text{H}^{\text{0}}\text{ =}{\text{ }\text{E}}_{\text{SCF}}\text{ + }\text{E}_{\text{vrt}}\text{ + R }\text{⋅}\text{ }\text{T}$ | (1) |
| --- | --- | --- |
|  | *E*_vrt_: sum of translational, rotational, and vibrational energy including zero-point energy |  |

Standard Gibbs free energy *G*^0^ obtained with help of the standard enthalpy *H*^0^ and standard entropy *S*^0^ (see Equation (2)).^[18]^

$$G^{0}\text{ }=\text{ }H^{0\text{ }}-\text{ }S^{0}\text{ }\text{⋅}\text{ }T$$

(2)

Solvation effects were investigated using CPCM, or the conductor like screening model (COSMO)^[19]^ or COSMO-RS and an assumed relative solvent permittivity *ε*_r_ pointing to infinity. For a relative solvent permittivity tending towards the infinite, the attractive and repulsive electrostatic forces *F*_solv_ between the charge carries vanish.^[20]^

Calculated species were investigated with help of Hess’ law of constant heat summation.^[21]^ Standard gas phase enthalpy of formation $\text{Δ}\text{H}_{\text{g,f}}^{\text{0}}$, standard gas phase Gibbs free energy of formation $\text{Δ}\text{G}_{\text{g,f}}^{\text{0}}$ and standard Gibbs free energy for the examined reactions of calculated species were obtained using following Equations (3), (4) and (5).^[21]^

$$\text{Δ}\text{H}_{\text{g,f}}^{\text{0}}\text{= }\sum\text{H}_{\text{g}}^{\text{0}}\left( Reduced \right) -\text{ }\sum\text{H}_{\text{g}}^{\text{0}}\text{(}\text{Oxidized}\text{)}$$

(3)

$$\text{Δ}\text{G}_{\text{g,f}}^{\text{0}}\text{= }\sum\text{G}_{\text{g}}^{\text{0}}\left( \text{Reduced} \right)\text{ }-\text{ }\sum\text{G}_{\text{g}}^{\text{0}}\text{(}\text{Oxidized}\text{)}$$

(4)

$$\text{Δ}\text{G}_{\text{solv}}^{\text{0}}\text{= }\sum\text{G}_{\text{solv}}^{\text{0}}\left( \text{Reduced} \right)\text{ }-\text{ }\sum\text{G}_{\text{solv}}^{\text{0}}\text{(}\text{Oxidized}\text{)}$$

(5)

The redox potentials were calculated following Equation (6).

$$\text{Δ}\text{G}_{\mathrm{total}}^{\text{0}}\text{= Δ}\text{G}_{\text{g,f}}^{\text{0}}+\text{Δ}\text{G}_{\text{solv}}^{\text{0}}$$

(6)

The redox potentials were calculated following Equation (7).^[22]^

$$\text{E }\text{= }-\frac{\text{Δ}\text{G}_{\mathrm{total}}^{\text{0}}}{nF}\text{ }$$

(7)

The redox potentials *vs.* SHE can be obtained following Equation (8).^[23,24]^

$$E_{\mathrm{SHE}}\text{= }\text{E }-\text{ }\text{4.28}$$

(8)

The redox potentials *vs.* Fc^+^/Fc can be obtained following Equation (9).^[23,24]^

$$E_{\mathrm{Fc}}\text{= }E_{\mathrm{SHE}}\text{ }-\text{ }\text{0.70}$$

(9)

# 9. Cartesian coordinates of optimized structures

**PNQ**

| C | 2.221988 | 2.760087 | -0.478071 |
| --- | --- | --- | --- |
| C | 2.721111 | 1.518852 | -0.068904 |
| C | 1.861964 | 0.440640 | 0.129873 |
| C | 0.476165 | 0.561149 | -0.071549 |
| C | -0.017001 | 1.822924 | -0.485719 |
| C | 0.856016 | 2.903354 | -0.683815 |
| C | -0.441571 | -0.578368 | 0.137462 |
| C | -1.835617 | -0.434007 | -0.069001 |
| C | -2.425498 | 0.855220 | -0.500147 |
| C | -1.461865 | 2.049809 | -0.723864 |
| C | 0.026617 | -1.839240 | 0.545531 |
| C | -0.844023 | -2.908838 | 0.740807 |
| C | -2.218916 | -2.753730 | 0.533951 |
| C | -2.705142 | -1.517032 | 0.130293 |
| O | -1.916308 | 3.124792 | -1.083814 |
| O | -3.622874 | 1.011042 | -0.683537 |
| H | 2.895901 | 3.602035 | -0.632747 |
| H | 3.790479 | 1.388019 | 0.097515 |
| H | 2.289136 | -0.507876 | 0.447925 |
| H | 0.422155 | 3.850658 | -1.002842 |
| H | 1.088695 | -2.000375 | 0.716751 |
| H | -0.444478 | -3.872384 | 1.057504 |
| H | -2.898025 | -3.591702 | 0.687493 |
| H | -3.767788 | -1.349501 | -0.043203 |

**PNQ^·‒^**

| C | 2.028565 | 2.427482 | -1.633411 |
| --- | --- | --- | --- |
| C | 2.613782 | 1.210337 | -1.233611 |
| C | 1.833476 | 0.242525 | -0.612652 |
| C | 0.458293 | 0.438991 | -0.365607 |
| C | -0.130273 | 1.674021 | -0.772860 |
| C | 0.680638 | 2.644910 | -1.402526 |
| C | -0.373566 | -0.566311 | 0.284963 |
| C | -1.758057 | -0.293196 | 0.499712 |
| C | -2.406176 | 0.970140 | 0.094000 |
| C | -1.556958 | 1.996445 | -0.569958 |
| C | 0.135217 | -1.809746 | 0.715983 |
| C | -0.670136 | -2.758220 | 1.334805 |
| C | -2.035687 | -2.484221 | 1.544396 |
| C | -2.559639 | -1.271035 | 1.130598 |
| O | -3.628623 | 1.163020 | 0.305212 |
| O | -2.027739 | 3.097682 | -0.946650 |
| H | 2.635274 | 3.193037 | -2.121927 |
| H | 3.674920 | 1.023339 | -1.408554 |
| H | 2.306408 | -0.692417 | -0.311296 |
| H | 0.186235 | 3.572103 | -1.695325 |
| H | 1.189419 | -2.042093 | 0.563512 |
| H | -0.241659 | -3.709702 | 1.655252 |
| H | -2.675071 | -3.224636 | 2.029990 |
| H | -3.610842 | -1.016853 | 1.272654 |

**PNQ^2‒^**

| C | 2.242417 | 2.734477 | -0.435907 |
| --- | --- | --- | --- |
| C | 2.770159 | 1.477624 | -0.020590 |
| C | 1.895805 | 0.412319 | 0.172643 |
| C | 0.500286 | 0.519616 | -0.024840 |
| C | -0.039513 | 1.799991 | -0.447910 |
| C | 0.882835 | 2.871694 | -0.637490 |
| C | -0.415121 | -0.570627 | 0.170371 |
| C | -1.827845 | -0.329714 | -0.066083 |
| C | -2.396874 | 0.947084 | -0.491208 |
| C | -1.454542 | 2.069028 | -0.692839 |
| C | -0.014362 | -1.862911 | 0.579423 |
| C | -0.912997 | -2.909323 | 0.764317 |
| C | -2.299402 | -2.674827 | 0.532880 |
| C | -2.723043 | -1.422640 | 0.132290 |
| O | -1.847845 | 3.227746 | -1.064693 |
| O | -3.655592 | 1.075630 | -0.677508 |
| H | 2.914785 | 3.585921 | -0.593696 |
| H | 3.844155 | 1.347509 | 0.145031 |
| H | 2.310798 | -0.548342 | 0.491244 |
| H | 0.434934 | 3.817006 | -0.956104 |
| H | 1.047703 | -2.053120 | 0.759520 |
| H | -0.556140 | -3.894002 | 1.081943 |
| H | -3.023233 | -3.486230 | 0.672987 |
| H | -3.776487 | -1.198208 | -0.056462 |

**ANQ**

| C | 2.414448 | 0.000000 | 0.328466 |
| --- | --- | --- | --- |
| C | 2.438059 | 0.000000 | -1.088496 |
| C | 1.188943 | 0.000000 | 0.966133 |
| C | 0.000000 | 0.000000 | 0.195082 |
| C | 1.274354 | 0.000000 | -1.847093 |
| C | 0.000000 | 0.000000 | -1.219029 |
| C | -1.188943 | 0.000000 | 0.966133 |
| C | -2.414448 | 0.000000 | 0.328466 |
| C | -2.438059 | 0.000000 | -1.088496 |
| C | -1.274354 | 0.000000 | -1.847093 |
| H | -3.341772 | 0.000000 | 0.901152 |
| H | -3.400992 | 0.000000 | -1.599276 |
| H | -1.337624 | 0.000000 | -2.936104 |
| H | 3.341772 | 0.000000 | 0.901152 |
| H | 3.400992 | 0.000000 | -1.599276 |
| H | 1.337624 | 0.000000 | -2.936104 |
| C | -0.796485 | 0.000000 | 2.397195 |
| C | 0.796485 | 0.000000 | 2.397195 |
| O | -1.493973 | 0.000000 | 3.390002 |
| O | 1.493973 | 0.000000 | 3.390002 |

**ANQ^·‒^**

| C | 2.385298 | 0.475521 | -0.023842 |
| --- | --- | --- | --- |
| C | 2.535114 | -0.938336 | 0.074652 |
| C | 1.453081 | -1.805791 | 0.097096 |
| C | 0.124149 | -1.281982 | 0.019490 |
| C | 0.011537 | 0.111130 | -0.076065 |
| C | 1.110040 | 1.010106 | -0.100235 |
| C | -1.103029 | -2.017128 | 0.028081 |
| C | -2.310034 | -1.338992 | -0.055829 |
| C | -2.389275 | 0.080794 | -0.151804 |
| C | -1.216776 | 0.817661 | -0.162316 |
| C | 0.579570 | 2.399951 | -0.209510 |
| C | -0.916596 | 2.276296 | -0.248877 |
| O | -1.764834 | 3.195217 | -0.336311 |
| O | 1.268908 | 3.446001 | -0.257244 |
| H | 3.262225 | 1.125573 | -0.038916 |
| H | 3.544064 | -1.354511 | 0.134765 |
| H | 1.612357 | -2.883705 | 0.173554 |
| H | -1.087033 | -3.106904 | 0.100386 |
| H | -3.238730 | -1.915416 | -0.048218 |
| H | -3.358948 | 0.578226 | -0.215989 |

**ANQ^2‒^**

| C | 2.406842 | 0.406107 | -0.002052 |
| --- | --- | --- | --- |
| C | 2.501203 | -0.995525 | -0.001221 |
| C | 1.361182 | -1.835718 | -0.000632 |
| C | 0.047002 | -1.261099 | -0.000887 |
| C | -0.014087 | 0.149494 | -0.001544 |
| C | 1.126497 | 1.008644 | -0.002015 |
| C | -1.212628 | -1.947181 | -0.000384 |
| C | -2.421046 | -1.208738 | -0.000758 |
| C | -2.448255 | 0.195835 | -0.001576 |
| C | -1.224738 | 0.906722 | -0.001884 |
| C | 0.651497 | 2.395745 | -0.002082 |
| C | -0.871686 | 2.329686 | -0.002405 |
| O | -1.672548 | 3.319072 | -0.002975 |
| O | 1.363633 | 3.450812 | -0.001650 |
| H | 3.308712 | 1.026361 | -0.002955 |
| H | 3.495184 | -1.461246 | -0.000883 |
| H | 1.482119 | -2.924792 | 0.000036 |
| H | -1.238933 | -3.042623 | 0.000260 |
| H | -3.371055 | -1.758635 | -0.000411 |
| H | -3.400395 | 0.735769 | -0.001942 |

**CDP**

| C | -0.778235 | -1.016823 | -0.210568 |
| --- | --- | --- | --- |
| C | 0.702841 | -1.027522 | 0.226688 |
| C | 1.229529 | 0.390077 | -0.086472 |
| C | 0.035147 | 1.307422 | 0.106519 |
| C | -1.250508 | 0.406573 | 0.026587 |
| O | -2.382847 | 0.816883 | 0.142241 |
| O | 0.023599 | 2.502928 | 0.292454 |
| H | -1.419756 | -1.744907 | 0.299618 |
| H | -0.865217 | -1.212924 | -1.293561 |
| H | 1.283159 | -1.811658 | -0.272295 |
| H | 0.766552 | -1.213722 | 1.307940 |
| H | 1.531936 | 0.467116 | -1.145470 |
| H | 2.087001 | 0.712016 | 0.516080 |

**CDP^·‒^**

| C | -0.692875 | -1.018212 | -0.048157 |
| --- | --- | --- | --- |
| C | 0.838315 | -1.051318 | 0.116898 |
| C | 1.288600 | 0.395914 | -0.157764 |
| C | 0.085934 | 1.270131 | 0.240415 |
| C | -1.101167 | 0.423122 | 0.305363 |
| O | -2.294890 | 0.756876 | 0.545349 |
| O | 0.173680 | 2.517940 | 0.411252 |
| H | -1.220094 | -1.747065 | 0.589691 |
| H | -0.985820 | -1.249584 | -1.093254 |
| H | 1.332602 | -1.794390 | -0.533834 |
| H | 1.086631 | -1.318411 | 1.156845 |
| H | 1.524176 | 0.542438 | -1.232288 |
| H | 2.193477 | 0.688731 | 0.400373 |

**CDP^2‒^**

| C | -0.342125 | 1.196150 | 0.132859 |
| --- | --- | --- | --- |
| C | -1.243724 | -0.000546 | -0.196160 |
| C | -0.342425 | -1.202226 | 0.115119 |
| C | 1.083084 | -0.715598 | -0.129596 |
| C | 1.083304 | 0.712790 | -0.119017 |
| O | 2.065914 | 1.569900 | -0.210101 |
| O | 2.065547 | -1.571548 | -0.233419 |
| H | -0.530454 | 1.507701 | 1.217554 |
| H | -0.570943 | 2.109759 | -0.460342 |
| H | -1.534118 | 0.007369 | -1.262934 |
| H | -2.194278 | -0.004946 | 0.419397 |
| H | -0.571517 | -2.106864 | -0.491558 |
| H | -0.530906 | -1.529789 | 1.195077 |

**BZ**

| C | -3.449005 | 0.203407 | -0.896085 |
| --- | --- | --- | --- |
| C | -4.160852 | -0.996414 | -0.815474 |
| C | -3.644943 | -2.074815 | -0.086205 |
| C | -2.419921 | -1.952660 | 0.560436 |
| C | -1.700384 | -0.747836 | 0.486328 |
| C | -2.221948 | 0.330586 | -0.247184 |
| C | -0.378355 | -0.669251 | 1.150005 |
| C | 0.378355 | 0.669251 | 1.150005 |
| O | 0.130127 | -1.598477 | 1.770717 |
| C | 1.700384 | 0.747836 | 0.486328 |
| O | -0.130127 | 1.598477 | 1.770717 |
| C | 2.419921 | 1.952660 | 0.560436 |
| C | 3.644943 | 2.074815 | -0.086205 |
| C | 4.160852 | 0.996414 | -0.815474 |
| C | 3.449005 | -0.203407 | -0.896085 |
| C | 2.221948 | -0.330586 | -0.247184 |
| H | -3.854135 | 1.041931 | -1.461951 |
| H | -5.121368 | -1.093083 | -1.322094 |
| H | -4.203357 | -3.008916 | -0.026294 |
| H | -1.993259 | -2.776341 | 1.132293 |
| H | -1.674518 | 1.270501 | -0.297491 |
| H | 1.993259 | 2.776341 | 1.132293 |
| H | 4.203357 | 3.008916 | -0.026294 |
| H | 5.121368 | 1.093083 | -1.322094 |
| H | 3.854135 | -1.041931 | -1.461951 |
| H | 1.674518 | -1.270501 | -0.297491 |

**BZ^·‒^**

| C | -3.903507 | 1.325906 | -0.027921 |
| --- | --- | --- | --- |
| C | -4.786589 | 0.240555 | -0.031080 |
| C | -4.259562 | -1.057501 | -0.038446 |
| C | -2.882670 | -1.257476 | -0.042544 |
| C | -1.974447 | -0.173252 | -0.039559 |
| C | -2.521041 | 1.130619 | -0.032130 |
| C | -0.516311 | -0.530182 | -0.043338 |
| C | 0.516216 | 0.529669 | -0.042921 |
| O | -0.221301 | -1.764861 | -0.046931 |
| C | 1.974342 | 0.172772 | -0.039216 |
| O | 0.221218 | 1.764362 | -0.045707 |
| C | 2.882487 | 1.257058 | -0.039918 |
| C | 4.259387 | 1.057174 | -0.036061 |
| C | 4.786494 | -0.240860 | -0.031093 |
| C | 3.903483 | -1.326280 | -0.030092 |
| C | 2.520999 | -1.131083 | -0.034159 |
| H | -4.297467 | 2.345145 | -0.022146 |
| H | -5.866494 | 0.401923 | -0.027878 |
| H | -4.932155 | -1.918832 | -0.040962 |
| H | -2.452802 | -2.259247 | -0.048014 |
| H | -1.832464 | 1.971321 | -0.030057 |
| H | 2.452547 | 2.258804 | -0.043527 |
| H | 4.931918 | 1.918556 | -0.036876 |
| H | 5.866411 | -0.402160 | -0.028042 |
| H | 4.297519 | -2.345500 | -0.026169 |
| H | 1.832467 | -1.971836 | -0.033754 |

**BZ^2‒^**

| C | -0.867188 | 1.104993 | 0.118465 |
| --- | --- | --- | --- |
| C | -1.473976 | -0.167931 | 0.185717 |
| C | -0.637046 | -1.297259 | 0.075637 |
| C | 0.735245 | -1.159063 | -0.092766 |
| C | 1.370865 | 0.121095 | -0.163903 |
| C | 0.507704 | 1.254043 | -0.050590 |
| C | 2.838522 | 0.135745 | -0.344977 |
| C | 3.543605 | 1.422551 | -0.426841 |
| O | 3.427964 | -1.021464 | -0.422497 |
| C | 5.011196 | 1.437131 | -0.608193 |
| O | 2.954213 | 2.579797 | -0.349065 |
| C | 5.646935 | 2.717241 | -0.679277 |
| C | 7.019194 | 2.855316 | -0.848057 |
| C | 7.855980 | 1.725919 | -0.958556 |
| C | 7.249068 | 0.453043 | -0.891391 |
| C | 5.874221 | 0.304110 | -0.721936 |
| H | -1.491961 | 2.002860 | 0.201118 |
| H | -2.554862 | -0.274948 | 0.318376 |
| H | -1.076026 | -2.301921 | 0.123729 |
| H | 1.398850 | -2.021975 | -0.179530 |
| H | 0.987439 | 2.231949 | -0.104174 |
| H | 4.983467 | 3.580230 | -0.592221 |
| H | 7.458254 | 3.859944 | -0.896125 |
| H | 8.936850 | 1.832848 | -1.091414 |
| H | 7.873714 | -0.444873 | -0.974464 |
| H | 5.394393 | -0.673752 | -0.668371 |

**PNQ dimer**

| C | -2.086555 | 0.351578 | 2.576427 |
| --- | --- | --- | --- |
| C | -2.240656 | 0.195636 | 1.050864 |
| C | -1.143518 | -0.738372 | 0.500753 |
| C | -1.386868 | -1.330255 | -0.906050 |
| C | -1.401775 | -0.292535 | -2.040752 |
| C | -2.482593 | 8.060989 | -2.632898 |
| C | -3.632112 | 7.287389 | -2.533155 |
| C | -3.592586 | 6.017018 | -1.938456 |
| C | -2.378983 | 5.496613 | -1.424359 |
| C | -1.228872 | 6.296994 | -1.538322 |
| C | -1.277403 | 7.556486 | -2.131378 |
| C | -4.855703 | 5.244493 | -1.869863 |
| C | -4.800564 | 3.825192 | -1.245140 |
| C | -3.498819 | 3.358485 | -0.712041 |
| C | -2.337266 | 4.162524 | -0.793647 |
| C | -3.450834 | 2.087363 | -0.122744 |
| C | -2.271283 | 1.563222 | 0.399331 |
| C | -1.123895 | 2.371085 | 0.334265 |
| C | -1.154858 | 3.633003 | -0.248209 |
| C | -0.357317 | -2.410089 | -1.175565 |
| C | -0.718528 | -3.751534 | -1.265449 |
| C | 0.228888 | -4.758731 | -1.500934 |
| C | 1.599493 | -4.441686 | -1.653320 |
| C | 1.955581 | -3.085078 | -1.561288 |
| C | 1.004302 | -2.097680 | -1.330590 |
| C | -0.254278 | -6.157030 | -1.584353 |
| C | 0.795510 | -7.267410 | -1.854958 |
| C | 2.212378 | -6.856942 | -1.997993 |
| C | 2.601395 | -5.497974 | -1.899299 |
| C | 3.165311 | -7.859657 | -2.234103 |
| C | 4.510424 | -7.542581 | -2.375005 |
| C | 4.907120 | -6.204092 | -2.277968 |
| C | 3.968140 | -5.201995 | -2.044537 |
| O | -5.815860 | 3.146612 | -1.211700 |
| O | -5.922129 | 5.673265 | -2.283560 |
| O | -1.428859 | -6.465610 | -1.450540 |
| O | 0.427179 | -8.429253 | -1.938159 |
| H | -2.144942 | -0.625718 | 3.075077 |
| H | -1.115195 | 0.802134 | 2.827580 |
| H | -2.872253 | 0.998274 | 2.988733 |
| H | -3.215903 | -0.281933 | 0.858407 |
| H | -0.170049 | -0.220248 | 0.518320 |
| H | -1.042240 | -1.581463 | 1.201865 |
| H | -2.372290 | -1.824956 | -0.886649 |
| H | -1.502873 | -0.793546 | -3.012759 |
| H | -2.235160 | 0.410992 | -1.934979 |
| H | -0.474204 | 0.297501 | -2.060315 |
| H | -2.517499 | 9.046628 | -3.096375 |
| H | -4.592311 | 7.638433 | -2.910836 |
| H | -0.273107 | 5.937968 | -1.161960 |
| H | -0.365095 | 8.149410 | -2.202955 |
| H | -4.377841 | 1.512134 | -0.094889 |
| H | -0.181005 | 2.006288 | 0.744462 |
| H | -0.233148 | 4.211306 | -0.272670 |
| H | -1.760066 | -4.058790 | -1.157000 |
| H | 2.994936 | -2.781811 | -1.672571 |
| H | 1.330340 | -1.058011 | -1.272643 |
| H | 2.809875 | -8.887810 | -2.301277 |
| H | 5.246898 | -8.324621 | -2.558113 |
| H | 5.958879 | -5.937091 | -2.385310 |
| H | 4.315757 | -4.173228 | -1.975136 |

**PNQ dimer^4‒^**

| C | -0.504964 | -1.169746 | 2.708215 |
| --- | --- | --- | --- |
| C | -1.168554 | -1.043957 | 1.331913 |
| C | -0.138327 | -0.471989 | 0.313176 |
| C | 0.835556 | -1.556481 | -0.236205 |
| C | 0.138885 | -2.346098 | -1.350384 |
| C | -9.165117 | 2.346316 | 1.607460 |
| C | -8.393975 | 2.196501 | 2.745210 |
| C | -7.062812 | 1.678872 | 2.718344 |
| C | -6.519830 | 1.298747 | 1.421950 |
| C | -7.332702 | 1.465933 | 0.279884 |
| C | -8.630671 | 1.975171 | 0.338182 |
| C | -6.346265 | 1.563010 | 3.981586 |
| C | -4.965573 | 1.032728 | 3.926051 |
| C | -4.441562 | 0.652784 | 2.609479 |
| C | -5.183761 | 0.767399 | 1.375691 |
| C | -3.121377 | 0.106871 | 2.545976 |
| C | -2.519694 | -0.329603 | 1.375758 |
| C | -3.272363 | -0.206475 | 0.166441 |
| C | -4.552417 | 0.328236 | 0.187579 |
| C | 2.228174 | -1.060619 | -0.627903 |
| C | 2.838380 | -1.360979 | -1.836124 |
| C | 4.192603 | -1.027876 | -2.152409 |
| C | 4.963170 | -0.321722 | -1.155525 |
| C | 4.322704 | -0.009094 | 0.067351 |
| C | 3.008589 | -0.358955 | 0.342808 |
| C | 4.719154 | -1.455127 | -3.453299 |
| C | 6.135841 | -1.144366 | -3.748798 |
| C | 6.881771 | -0.420425 | -2.728083 |
| C | 6.334228 | 0.002114 | -1.446802 |
| C | 8.248723 | -0.101489 | -2.993892 |
| C | 9.049264 | 0.581808 | -2.097428 |
| C | 8.509764 | 0.998864 | -0.844745 |
| C | 7.177373 | 0.700873 | -0.555757 |
| O | -4.253684 | 0.896584 | 4.979461 |
| O | -6.898787 | 1.908908 | 5.088876 |
| O | 3.979078 | -2.081838 | -4.286653 |
| O | 6.693400 | -1.492244 | -4.852954 |
| H | 0.457648 | -1.697880 | 2.627230 |
| H | -0.310430 | -0.173249 | 3.132838 |
| H | -1.143835 | -1.711169 | 3.420511 |
| H | -1.387643 | -2.076660 | 0.988136 |
| H | -0.663302 | -0.007611 | -0.535844 |
| H | 0.429089 | 0.335912 | 0.800343 |
| H | 0.999657 | -2.265948 | 0.602048 |
| H | 0.727375 | -3.222316 | -1.658001 |
| H | -0.854010 | -2.688307 | -1.019760 |
| H | 0.002558 | -1.713583 | -2.240652 |
| H | -10.183820 | 2.750886 | 1.677447 |
| H | -8.763965 | 2.468765 | 3.738650 |
| H | -6.926881 | 1.187916 | -0.697596 |
| H | -9.221432 | 2.089806 | -0.577250 |
| H | -2.622653 | 0.036991 | 3.514919 |
| H | -2.841854 | -0.543306 | -0.780828 |
| H | -5.096595 | 0.398735 | -0.759297 |
| H | 2.318764 | -1.906788 | -2.626425 |
| H | 4.886874 | 0.520292 | 0.841455 |
| H | 2.573798 | -0.105986 | 1.313869 |
| H | 8.621303 | -0.437821 | -3.966551 |
| H | 10.095030 | 0.806148 | -2.347115 |
| H | 9.123193 | 1.547344 | -0.121520 |
| H | 6.768636 | 1.027599 | 0.405332 |

**PNQ dimer *meta***

| C | 2.958968 | -1.720924 | 1.666235 |
| --- | --- | --- | --- |
| C | 3.839418 | -1.713693 | 0.592926 |
| C | 3.918134 | -0.586743 | -0.229155 |
| C | 3.069276 | 0.540171 | -0.040351 |
| C | 2.242071 | 0.566854 | 1.113864 |
| C | 2.214716 | -0.575536 | 1.933494 |
| C | 4.999240 | -0.572315 | -1.245471 |
| C | 5.404893 | 0.818887 | -1.776358 |
| C | 4.302269 | 1.803203 | -1.831373 |
| C | 3.121522 | 1.610532 | -1.067868 |
| C | 4.407080 | 2.854664 | -2.751318 |
| C | 3.328876 | 3.701802 | -2.984252 |
| C | 2.128233 | 3.466668 | -2.310063 |
| C | 2.029398 | 2.440424 | -1.369453 |
| O | 5.591650 | -1.572039 | -1.621356 |
| O | 6.537641 | 1.026548 | -2.181059 |
| C | 1.464676 | 1.790808 | 1.589044 |
| C | -0.071096 | 1.708487 | 1.360680 |
| C | -0.851529 | 0.454313 | 1.819781 |
| C | -4.499338 | 1.236216 | 1.024675 |
| C | -4.862244 | 0.188048 | 0.187446 |
| C | -3.897989 | -0.735450 | -0.226859 |
| C | -2.533385 | -0.601090 | 0.147011 |
| C | -2.199038 | 0.384309 | 1.108730 |
| C | -3.191476 | 1.296893 | 1.502464 |
| C | -4.365611 | -1.927064 | -0.978430 |
| C | -3.386798 | -3.125691 | -1.034767 |
| C | -1.953147 | -2.766057 | -0.986339 |
| C | -1.548453 | -1.495338 | -0.505020 |
| C | -1.023970 | -3.653914 | -1.545467 |
| C | 0.306920 | -3.279199 | -1.700094 |
| C | 0.697353 | -1.991744 | -1.322528 |
| C | -0.215943 | -1.117862 | -0.733352 |
| O | -3.808186 | -4.261542 | -1.186063 |
| O | -5.473257 | -2.018391 | -1.484359 |
| C | -1.062573 | 0.378204 | 3.343084 |
| C | 1.851311 | 2.160160 | 3.034266 |
| H | 2.883474 | -2.588729 | 2.320828 |
| H | 4.501027 | -2.552960 | 0.380002 |
| H | 1.598391 | -0.561524 | 2.830915 |
| H | 5.344828 | 2.960478 | -3.297078 |
| H | 3.407111 | 4.511037 | -3.709733 |
| H | 1.252836 | 4.081512 | -2.520650 |
| H | 1.068415 | 2.269273 | -0.894943 |
| H | 1.796829 | 2.642713 | 0.980638 |
| H | -0.525154 | 2.598504 | 1.827175 |
| H | -0.264555 | 1.825664 | 0.283237 |
| H | -0.281070 | -0.436777 | 1.531138 |
| H | -5.236157 | 1.972051 | 1.346429 |
| H | -5.889277 | 0.043149 | -0.147076 |
| H | -2.932820 | 2.070189 | 2.226840 |
| H | -1.387478 | -4.624187 | -1.884448 |
| H | 1.026961 | -3.964543 | -2.146417 |
| H | 1.720767 | -1.658117 | -1.487913 |
| H | 0.119741 | -0.117294 | -0.479790 |
| H | -1.710085 | -0.470347 | 3.600191 |
| H | -0.112403 | 0.244167 | 3.874238 |
| H | -1.528727 | 1.290160 | 3.740219 |
| H | 1.314482 | 3.067400 | 3.344688 |
| H | 2.928519 | 2.363394 | 3.098717 |
| H | 1.620505 | 1.374285 | 3.762059 |

**PNQ dimer *meta*^4‒^**

| C | 3.252510 | -0.867522 | 0.480758 |
| --- | --- | --- | --- |
| C | 4.408118 | -0.122657 | 0.529381 |
| C | 4.436267 | 1.254502 | 0.185164 |
| C | 3.207237 | 1.894470 | -0.290622 |
| C | 1.977131 | 1.117813 | -0.201988 |
| C | 2.035215 | -0.229613 | 0.144951 |
| C | 5.749456 | 1.901208 | 0.346165 |
| C | 5.848490 | 3.322568 | 0.002141 |
| C | 4.667148 | 3.910941 | -0.609460 |
| C | 3.373143 | 3.241184 | -0.794900 |
| C | 4.813582 | 5.247321 | -1.087644 |
| C | 3.824832 | 5.922841 | -1.773930 |
| C | 2.592933 | 5.258242 | -2.024180 |
| C | 2.404979 | 3.967823 | -1.536568 |
| O | 6.760291 | 1.238500 | 0.775906 |
| O | 6.929507 | 3.999979 | 0.173381 |
| C | 0.566669 | 1.703476 | -0.386707 |
| C | -0.651241 | 0.726543 | -0.519812 |
| C | -1.299521 | -0.127345 | 0.638965 |
| C | -4.802209 | 1.374294 | 0.404815 |
| C | -5.627396 | 0.280080 | 0.258118 |
| C | -5.128508 | -1.049211 | 0.291806 |
| C | -3.704379 | -1.262303 | 0.569392 |
| C | -2.845057 | -0.103760 | 0.579789 |
| C | -3.408434 | 1.174905 | 0.526099 |
| C | -6.123780 | -2.098599 | 0.034306 |
| C | -5.650291 | -3.485767 | 0.029830 |
| C | -4.277677 | -3.693856 | 0.474477 |
| C | -3.315371 | -2.638082 | 0.789568 |
| C | -3.868362 | -5.049089 | 0.650689 |
| C | -2.648012 | -5.404288 | 1.190833 |
| C | -1.754066 | -4.382070 | 1.606604 |
| C | -2.103264 | -3.049404 | 1.401045 |
| O | -6.412562 | -4.475790 | -0.277607 |
| O | -7.352591 | -1.797827 | -0.192257 |
| C | -0.838966 | 0.228493 | 2.065370 |
| C | 0.304782 | 2.837906 | 0.625719 |
| H | 3.252437 | -1.932313 | 0.737963 |
| H | 5.370543 | -0.539872 | 0.838412 |
| H | 1.113040 | -0.806918 | 0.183271 |
| H | 5.793938 | 5.691091 | -0.886393 |
| H | 3.990060 | 6.947521 | -2.133007 |
| H | 1.792533 | 5.743100 | -2.593014 |
| H | 1.456561 | 3.495607 | -1.776581 |
| H | 0.544069 | 2.174631 | -1.382682 |
| H | -1.453541 | 1.355082 | -0.931626 |
| H | -0.402023 | 0.018728 | -1.327148 |
| H | -0.977621 | -1.157022 | 0.451421 |
| H | -5.207368 | 2.393856 | 0.385259 |
| H | -6.705428 | 0.365207 | 0.093913 |
| H | -2.747634 | 2.044679 | 0.552751 |
| H | -4.616690 | -5.791662 | 0.356689 |
| H | -2.381531 | -6.461386 | 1.321589 |
| H | -0.804702 | -4.623940 | 2.093415 |
| H | -1.417484 | -2.298184 | 1.783381 |
| H | -1.248279 | -0.505421 | 2.777176 |
| H | 0.257581 | 0.226086 | 2.148830 |
| H | -1.216560 | 1.214527 | 2.367418 |
| H | -0.730277 | 3.207411 | 0.539638 |
| H | 0.992673 | 3.675034 | 0.452315 |
| H | 0.458688 | 2.489038 | 1.653464 |

**PNQ dimer *ortho***

| C | -1.504437 | -3.054246 | -2.614716 |
| --- | --- | --- | --- |
| C | -1.845287 | -1.752168 | -2.236315 |
| C | -2.184417 | -1.525032 | -0.877341 |
| C | -2.139456 | -2.575060 | 0.077888 |
| C | -1.805380 | -3.862891 | -0.356631 |
| C | -1.496680 | -4.098990 | -1.691306 |
| C | -2.558224 | -0.181995 | -0.377108 |
| C | -2.146289 | 0.123502 | 1.075255 |
| C | -2.295939 | -0.991728 | 2.017907 |
| C | -2.374567 | -2.310850 | 1.515182 |
| C | -2.376375 | -0.727590 | 3.390876 |
| C | -2.577675 | -1.766841 | 4.291145 |
| C | -2.694852 | -3.073745 | 3.806137 |
| C | -2.589340 | -3.341922 | 2.441273 |
| O | -1.749137 | 1.244018 | 1.376202 |
| O | -3.155270 | 0.678565 | -1.011620 |
| C | -1.756990 | -0.612034 | -3.240168 |
| C | -0.600000 | 0.365515 | -2.882084 |
| C | 0.809892 | -0.224627 | -2.592464 |
| C | 2.243956 | 1.827972 | -2.583309 |
| C | 1.554902 | 0.865214 | -1.836593 |
| C | 1.492539 | 1.025348 | -0.418567 |
| C | 2.018212 | 2.205514 | 0.190255 |
| C | 2.700508 | 3.132057 | -0.608109 |
| C | 2.829969 | 2.935191 | -1.978143 |
| C | 0.976869 | -0.042093 | 0.467422 |
| C | 1.083777 | 0.141231 | 2.007588 |
| C | 1.414467 | 1.476255 | 2.521894 |
| C | 1.844500 | 2.483550 | 1.633323 |
| C | 1.267399 | 1.728770 | 3.893008 |
| C | 1.522383 | 2.993543 | 4.405951 |
| C | 1.922569 | 4.012074 | 3.532978 |
| C | 2.081224 | 3.760870 | 2.172009 |
| O | 0.849622 | -0.820050 | 2.726046 |
| O | 0.496744 | -1.106340 | 0.090891 |
| C | 1.549313 | -0.710742 | -3.847323 |
| C | -1.734740 | -1.063423 | -4.705332 |
| H | -1.230242 | -3.259517 | -3.647613 |
| H | -1.742903 | -4.682495 | 0.356074 |
| H | -1.223035 | -5.104262 | -2.013462 |
| H | -2.289662 | 0.308029 | 3.719022 |
| H | -2.653593 | -1.567245 | 5.359696 |
| H | -2.868021 | -3.897288 | 4.499608 |
| H | -2.679651 | -4.372425 | 2.101873 |
| H | -2.667608 | -0.012480 | -3.109400 |
| H | -0.513546 | 1.108967 | -3.692534 |
| H | -0.910686 | 0.936764 | -1.998860 |
| H | 0.681790 | -1.077272 | -1.920325 |
| H | 2.318733 | 1.710461 | -3.663022 |
| H | 3.153211 | 4.012527 | -0.157952 |
| H | 3.377018 | 3.661134 | -2.580744 |
| H | 0.938663 | 0.906413 | 4.527951 |
| H | 1.398972 | 3.196117 | 5.469655 |
| H | 2.105451 | 5.016904 | 3.914953 |
| H | 2.370110 | 4.585846 | 1.524062 |
| H | 2.610026 | -0.899623 | -3.631527 |
| H | 1.117057 | -1.655052 | -4.199871 |
| H | 1.491827 | 0.002768 | -4.682100 |
| H | -1.768480 | -0.180226 | -5.357639 |
| H | -2.610832 | -1.684041 | -4.939935 |
| H | -0.837100 | -1.632009 | -4.978119 |

**PNQ dimer *ortho*^4‒^**

| C | -0.286942 | -1.743583 | 1.950712 |
| --- | --- | --- | --- |
| C | -1.573275 | -1.317649 | 1.642077 |
| C | -2.625079 | -1.407459 | 2.653032 |
| C | -2.226861 | -1.860416 | 3.988617 |
| C | -0.893263 | -2.271345 | 4.219411 |
| C | 0.067741 | -2.239354 | 3.227731 |
| C | -4.033219 | -1.094368 | 2.351699 |
| C | -5.023822 | -1.100507 | 3.464361 |
| C | -4.567281 | -1.518448 | 4.772020 |
| C | -3.196009 | -1.903863 | 5.056267 |
| C | -5.514341 | -1.575382 | 5.840407 |
| C | -5.179810 | -1.974655 | 7.119851 |
| C | -3.831176 | -2.348917 | 7.405944 |
| C | -2.889137 | -2.307377 | 6.376219 |
| O | -6.249562 | -0.782887 | 3.262268 |
| O | -4.440696 | -0.830279 | 1.172480 |
| C | -1.803253 | -0.807549 | 0.203016 |
| C | -0.622604 | 0.045690 | -0.336357 |
| C | -0.749495 | 0.603866 | -1.776228 |
| C | 1.392162 | 1.822817 | -1.334582 |
| C | 0.520525 | 1.313497 | -2.289493 |
| C | 0.803657 | 1.522333 | -3.711734 |
| C | 1.983180 | 2.325720 | -4.043029 |
| C | 2.821604 | 2.801150 | -3.007563 |
| C | 2.555387 | 2.554676 | -1.675689 |
| C | -0.029482 | 0.934125 | -4.776125 |
| C | 0.311408 | 1.221742 | -6.198816 |
| C | 1.458486 | 2.061534 | -6.464403 |
| C | 2.296959 | 2.621623 | -5.419766 |
| C | 1.790632 | 2.358095 | -7.822015 |
| C | 2.862569 | 3.154533 | -8.175811 |
| C | 3.683091 | 3.718494 | -7.151454 |
| C | 3.381340 | 3.437386 | -5.817526 |
| O | -0.381960 | 0.727858 | -7.157511 |
| O | -1.032242 | 0.184800 | -4.533695 |
| C | -1.946995 | 1.560885 | -1.912721 |
| C | -2.135094 | -2.002355 | -0.708708 |
| H | 0.486289 | -1.686461 | 1.183561 |
| H | -0.613871 | -2.641665 | 5.209221 |
| H | 1.093800 | -2.565373 | 3.430493 |
| H | -6.532948 | -1.278688 | 5.571278 |
| H | -5.941006 | -2.000389 | 7.911627 |
| H | -3.532960 | -2.662590 | 8.413012 |
| H | -1.860067 | -2.585965 | 6.621458 |
| H | -2.716144 | -0.200057 | 0.242126 |
| H | -0.486389 | 0.871882 | 0.383309 |
| H | 0.314804 | -0.533541 | -0.310719 |
| H | -0.933940 | -0.214385 | -2.484919 |
| H | 1.180170 | 1.652925 | -0.279446 |
| H | 3.724269 | 3.359758 | -3.268264 |
| H | 3.229950 | 2.917399 | -0.892326 |
| H | 1.129571 | 1.907478 | -8.568987 |
| H | 3.079143 | 3.358595 | -9.233383 |
| H | 4.531649 | 4.366566 | -7.399346 |
| H | 4.010784 | 3.896212 | -5.049467 |
| H | -2.053181 | 1.863574 | -2.962380 |
| H | -2.881760 | 1.078992 | -1.589336 |
| H | -1.791600 | 2.457246 | -1.286417 |
| H | -2.325634 | -1.677808 | -1.742418 |
| H | -3.036192 | -2.497271 | -0.320721 |
| H | -1.301661 | -2.726273 | -0.723904 |

**PNQ dimer *para***

| C | 2.335367 | 2.418465 | 1.158151 |
| --- | --- | --- | --- |
| C | 3.237753 | 2.429208 | 0.107222 |
| C | 2.945505 | 3.102556 | -1.089305 |
| C | 1.718412 | 3.792911 | -1.234537 |
| C | 0.823641 | 3.762214 | -0.153087 |
| C | 1.102247 | 3.081434 | 1.034721 |
| C | 3.950967 | 3.065489 | -2.173067 |
| C | 3.624030 | 3.830956 | -3.481731 |
| C | 2.317350 | 4.527712 | -3.564933 |
| C | 1.398957 | 4.511234 | -2.486297 |
| C | 2.013955 | 5.210469 | -4.752429 |
| C | 0.807513 | 5.884260 | -4.896664 |
| C | -0.107176 | 5.875509 | -3.838138 |
| C | 0.185674 | 5.200475 | -2.655057 |
| O | 5.011722 | 2.465435 | -2.079450 |
| O | 4.439016 | 3.836825 | -4.391337 |
| C | 0.101803 | 3.087628 | 2.171098 |
| C | -0.317039 | 1.680371 | 2.645177 |
| C | -1.201437 | 0.869072 | 1.670293 |
| C | -3.239719 | -0.318675 | 2.608335 |
| C | -3.819656 | -1.369928 | 3.304465 |
| C | -3.033072 | -2.405572 | 3.828266 |
| C | -1.627574 | -2.392133 | 3.648132 |
| C | -1.071237 | -1.318744 | 2.938354 |
| C | -1.850638 | -0.280251 | 2.416355 |
| C | -3.713513 | -3.493160 | 4.564603 |
| C | -2.829268 | -4.620962 | 5.157517 |
| C | -1.367020 | -4.546782 | 4.921972 |
| C | -0.786437 | -3.478614 | 4.194478 |
| C | -0.567711 | -5.575319 | 5.443760 |
| C | 0.808817 | -5.569965 | 5.257655 |
| C | 1.393573 | -4.521629 | 4.538873 |
| C | 0.607988 | -3.495879 | 4.017881 |
| O | -3.355003 | -5.523673 | 5.790530 |
| O | -4.924530 | -3.536312 | 4.724201 |
| C | 0.654478 | 3.903900 | 3.354879 |
| C | -0.466185 | 0.357738 | 0.418975 |
| H | 2.581792 | 1.887973 | 2.078499 |
| H | 4.197017 | 1.916186 | 0.176523 |
| H | -0.136262 | 4.272474 | -0.224923 |
| H | 2.756354 | 5.190863 | -5.550139 |
| H | 0.576498 | 6.412604 | -5.821302 |
| H | -1.058547 | 6.399508 | -3.933887 |
| H | -0.550958 | 5.217795 | -1.854496 |
| H | -0.807039 | 3.596892 | 1.807862 |
| H | 0.578273 | 1.093805 | 2.908742 |
| H | -0.878942 | 1.806438 | 3.583647 |
| H | -2.015889 | 1.534648 | 1.336921 |
| H | -3.860942 | 0.483174 | 2.205787 |
| H | -4.896666 | -1.421234 | 3.464505 |
| H | 0.005219 | -1.278744 | 2.780968 |
| H | -1.066446 | -6.372098 | 5.995372 |
| H | 1.425074 | -6.371416 | 5.664339 |
| H | 2.472301 | -4.502293 | 4.381070 |
| H | 1.100892 | -2.700020 | 3.463358 |
| H | -0.099174 | 3.991551 | 4.149506 |
| H | 0.941785 | 4.915057 | 3.037666 |
| H | 1.544502 | 3.423277 | 3.785536 |
| H | -1.136693 | -0.263225 | -0.189939 |
| H | -0.106345 | 1.182886 | -0.205579 |
| H | 0.405148 | -0.255465 | 0.689203 |

**PNQ dimer *para*^4‒^**

| C | 3.117817 | 0.209300 | 0.164157 |
| --- | --- | --- | --- |
| C | 4.349187 | 0.308455 | -0.455780 |
| C | 4.860237 | 1.530895 | -0.980456 |
| C | 4.003355 | 2.693147 | -0.883590 |
| C | 2.747741 | 2.550868 | -0.241029 |
| C | 2.278676 | 1.355604 | 0.298741 |
| C | 6.207038 | 1.530206 | -1.559897 |
| C | 6.720344 | 2.815697 | -2.078335 |
| C | 5.831558 | 3.966831 | -1.983373 |
| C | 4.491993 | 3.939203 | -1.410918 |
| C | 6.304138 | 5.218451 | -2.483745 |
| C | 5.548825 | 6.375493 | -2.448767 |
| C | 4.235604 | 6.344616 | -1.893697 |
| C | 3.746036 | 5.138435 | -1.394566 |
| O | 6.904329 | 0.457159 | -1.597387 |
| O | 7.894617 | 2.925378 | -2.589735 |
| C | 1.040892 | 1.351586 | 1.187119 |
| C | 0.025591 | 0.189835 | 1.040653 |
| C | -0.891748 | 0.237935 | -0.202041 |
| C | -3.397679 | 0.224130 | -0.658775 |
| C | -4.667915 | -0.300308 | -0.520466 |
| C | -4.947590 | -1.436031 | 0.292091 |
| C | -3.827166 | -2.036359 | 0.984304 |
| C | -2.535595 | -1.469592 | 0.811516 |
| C | -2.281983 | -0.354618 | 0.016540 |
| C | -6.336054 | -1.901278 | 0.373569 |
| C | -6.606168 | -3.071807 | 1.230558 |
| C | -5.463369 | -3.659110 | 1.918825 |
| C | -4.090980 | -3.177499 | 1.820855 |
| C | -5.701801 | -4.791650 | 2.756142 |
| C | -4.698842 | -5.429553 | 3.461451 |
| C | -3.357692 | -4.955717 | 3.365492 |
| C | -3.091204 | -3.853021 | 2.555211 |
| O | -7.788872 | -3.558638 | 1.372360 |
| O | -7.265645 | -1.304045 | -0.276828 |
| C | 1.493425 | 1.431167 | 2.665013 |
| C | -0.198439 | -0.386539 | -1.429711 |
| H | 2.781037 | -0.755972 | 0.556306 |
| H | 5.006181 | -0.558980 | -0.563426 |
| H | 2.111823 | 3.435557 | -0.127417 |
| H | 7.315311 | 5.196117 | -2.902135 |
| H | 5.953814 | 7.314303 | -2.849617 |
| H | 3.615077 | 7.246592 | -1.864926 |
| H | 2.734635 | 5.120881 | -0.978385 |
| H | 0.487172 | 2.284650 | 0.970482 |
| H | 0.562038 | -0.774089 | 1.079952 |
| H | -0.624265 | 0.204501 | 1.930775 |
| H | -1.045670 | 1.309364 | -0.435798 |
| H | -3.232106 | 1.109152 | -1.286171 |
| H | -5.532363 | 0.141265 | -1.024572 |
| H | -1.693720 | -1.930486 | 1.335124 |
| H | -6.744862 | -5.120529 | 2.800558 |
| H | -4.930984 | -6.297197 | 4.093597 |
| H | -2.546747 | -5.444270 | 3.915659 |
| H | -2.059800 | -3.495894 | 2.488513 |
| H | 0.632437 | 1.535930 | 3.348486 |
| H | 2.175814 | 2.281182 | 2.811872 |
| H | 2.040174 | 0.516394 | 2.943287 |
| H | -0.834493 | -0.286238 | -2.322277 |
| H | 0.776564 | 0.083770 | -1.626023 |
| H | -0.036108 | -1.462101 | -1.257161 |
|  |  |  |  |

# 10. X-Ray data

Crystals were obtained at room temperature by slow evaporation of the solvent chloroform. A yellow, block-shaped crystal was mounted on a MiTeGen micromount with perfluoroether oil. Data for Werz_ND165_a were collected from a shock-cooled single crystal at 100(2) K on a Bruker D8 VENTURE dual wavelength Mo/Cu three-circle diffractometer with a microfocus sealed X-ray tube using a mirror optics as monochromator and a Bruker PHOTON III detector. The diffractometer was equipped with an Oxford Cryostream 800 low temperature device and used Mo*K_α_* radiation (λ = 0.71073 Å). All data were integrated with SAINT V8.42 and a multi-scan absorption correction using SADABS 2016/2 was applied.^[25]^ The structure was solved by direct methods with SHELXT and refined by full-matrix least-squares methods against *F*^2^ using SHELXL-2019/2.^[26]^ All non-hydrogen atoms were refined with anisotropic displacement parameters. All hydrogen atoms were refined isotropic on calculated positions using a riding model with their *U*_iso_ values constrained to 1.5 times the *U*_eq_ of their pivot atoms for terminal sp^3^ carbon atoms and 1.2 times for all other carbon atoms. Crystallographic data for the structures reported in this paper have been deposited with the Cambridge Crystallographic Data Centre.^[27]^ CCDC 2526325 contain the supplementary crystallographic data for this paper. These data can be obtained free of charge from The Cambridge Crystallographic Data Centre via www.ccdc.cam.ac.uk/‌structures. This report and the CIF file were generated using FinalCif.^[28]^
 **Table 1 Crystal data and structure refinement for Werz_ND165_a**

| CCDC number | 2526325 |
| --- | --- |
| Empirical formula | C_28_H_38_O_4_Si_2_ |
| Formula weight | 494.76 |
| Temperature [K] | 100(2) |
| Crystal system | triclinic |
| Space group (number) | $P\overline{1}$ (2) |
| *a* [Å] | 8.6394(19) |
| *b* [Å] | 9.9103(15) |
| *c* [Å] | 16.688(4) |
| α [°] | 73.819(13) |
| β [°] | 89.842(10) |
| γ [°] | 84.580(16) |
| Volume [Å^3^] | 1365.7(5) |
| *Z* | 2 |
| *ρ*_calc_ [gcm^−3^] | 1.203 |
| *μ* [mm^−1^] | 0.160 |
| *F*(000) | 532 |
| Crystal size [mm^3^] | 0.072×0.322×0.342 |
| Crystal colour | yellow |
| Crystal shape | block |
| Radiation | Mo*K_α_* (λ=0.71073 Å) |
| 2θ range [°] | 4.30 to 58.41 (0.73 Å) |
| Index ranges | −11 ≤ h ≤ 11 −13 ≤ k ≤ 13 −22 ≤ l ≤ 22 |
| Reflections collected | 81001 |
| Independent reflections | 7387  *R*_int_ = 0.0632 *R*_sigma_ = 0.0259 |
| Completeness to  θ = 25.242° | 100.0 % |
| Data / Restraints / Parameters | 7387 / 0 / 317 |
| Absorption correction T_min_/T_max_ (method) | 0.7011 / 0.7458  (multi-scan) |
| Goodness-of-fit on *F*^2^ | 1.041 |
| Final *R* indexes  [*I*≥2σ(*I*)] | *R*_1_ = 0.0368 w*R*_2_ = 0.0944 |
| Final *R* indexes  [all data] | *R*_1_ = 0.0491 w*R*_2_ = 0.1020 |
| Largest peak/hole [eÅ^−3^] | 0.37/−0.25 |

**Table 1 Atomic coordinates and *U_eq_* [Å^2^] for Werz_ND165_a**

| **Atom** | ***x*** | ***y*** | ***z*** | ***U*_eq_** |
| --- | --- | --- | --- | --- |
| Si1 | 0.83603(4) | 0.70656(4) | 0.62585(2) | 0.02003(9) |
| Si2 | 0.59051(4) | 0.99822(3) | 0.76790(2) | 0.01780(8) |
| O1 | 0.83229(10) | 0.70053(9) | 0.72726(5) | 0.01904(18) |
| O2 | 0.54823(10) | 0.85851(8) | 0.73629(5) | 0.01832(18) |
| O3 | 1.12220(13) | 0.09464(11) | 0.91201(7) | 0.0376(3) |
| O4 | −0.07049(11) | 0.71633(11) | 0.92162(6) | 0.0296(2) |
| C1 | 0.71595(14) | 0.64385(12) | 0.77876(7) | 0.0173(2) |
| C2 | 0.74654(14) | 0.50086(12) | 0.83078(7) | 0.0175(2) |
| C3 | 0.89412(15) | 0.42618(13) | 0.83145(8) | 0.0202(2) |
| H3 | 0.973331 | 0.471018 | 0.797449 | 0.024 |
| C4 | 0.92523(15) | 0.28903(13) | 0.88069(8) | 0.0209(2) |
| C5 | 0.80952(16) | 0.22135(13) | 0.93186(8) | 0.0215(3) |
| H5 | 0.830542 | 0.126654 | 0.965398 | 0.026 |
| C6 | 0.66637(15) | 0.29311(13) | 0.93293(8) | 0.0205(2) |
| H6 | 0.589217 | 0.247375 | 0.968204 | 0.025 |
| C7 | 0.63023(15) | 0.43385(12) | 0.88280(7) | 0.0179(2) |
| C8 | 0.47954(15) | 0.51160(12) | 0.88314(7) | 0.0178(2) |
| C9 | 0.35482(15) | 0.45071(13) | 0.93105(8) | 0.0210(2) |
| H9 | 0.369032 | 0.354763 | 0.963364 | 0.025 |
| C10 | 0.21420(15) | 0.52720(13) | 0.93177(8) | 0.0213(2) |
| H10 | 0.132024 | 0.484300 | 0.964068 | 0.026 |
| C11 | 0.19233(15) | 0.66964(13) | 0.88438(8) | 0.0210(2) |
| C12 | 0.31106(14) | 0.73126(13) | 0.83649(8) | 0.0196(2) |
| H12 | 0.294661 | 0.827228 | 0.804403 | 0.024 |
| C13 | 0.45597(14) | 0.65459(12) | 0.83430(7) | 0.0179(2) |
| C14 | 0.57816(14) | 0.72027(12) | 0.78297(7) | 0.0173(2) |
| C15 | 1.08185(17) | 0.21694(14) | 0.87786(9) | 0.0267(3) |
| H15 | 1.157377 | 0.271732 | 0.846387 | 0.032 |
| C16 | 0.04578(16) | 0.75807(14) | 0.88668(9) | 0.0253(3) |
| H16 | 0.042650 | 0.855818 | 0.858365 | 0.030 |
| C17 | 0.7549(2) | 0.54636(15) | 0.61214(9) | 0.0325(3) |
| H17A | 0.754550 | 0.549857 | 0.552874 | 0.049 |
| H17B | 0.648346 | 0.543306 | 0.632334 | 0.049 |
| H17C | 0.819302 | 0.461739 | 0.644015 | 0.049 |
| C18 | 0.71728(17) | 0.86678(15) | 0.56284(8) | 0.0268(3) |
| H18A | 0.725371 | 0.871996 | 0.503491 | 0.040 |
| H18B | 0.755445 | 0.950859 | 0.572822 | 0.040 |
| H18C | 0.608330 | 0.862041 | 0.578961 | 0.040 |
| C19 | 1.04687(16) | 0.71344(14) | 0.59678(9) | 0.0260(3) |
| C20 | 1.14876(19) | 0.58151(17) | 0.64641(12) | 0.0415(4) |
| H20A | 1.106989 | 0.497021 | 0.639111 | 0.062 |
| H20B | 1.149127 | 0.577749 | 0.705718 | 0.062 |
| H20C | 1.255246 | 0.585496 | 0.626112 | 0.062 |
| C21 | 1.0585(2) | 0.72516(16) | 0.50295(10) | 0.0359(4) |
| H21A | 0.996501 | 0.810995 | 0.470702 | 0.054 |
| H21B | 1.018960 | 0.642514 | 0.491788 | 0.054 |
| H21C | 1.167400 | 0.729430 | 0.486695 | 0.054 |
| C22 | 1.10907(18) | 0.84308(16) | 0.61388(10) | 0.0317(3) |
| H22A | 1.219093 | 0.845104 | 0.599601 | 0.048 |
| H22B | 1.098075 | 0.837852 | 0.673108 | 0.048 |
| H22C | 1.049750 | 0.929054 | 0.579864 | 0.048 |
| C23 | 0.55939(18) | 0.95698(14) | 0.88200(8) | 0.0277(3) |
| H23A | 0.595274 | 1.031902 | 0.903053 | 0.041 |
| H23B | 0.618084 | 0.866740 | 0.910336 | 0.041 |
| H23C | 0.448399 | 0.950520 | 0.892820 | 0.041 |
| C24 | 0.79585(16) | 1.03400(15) | 0.74511(10) | 0.0281(3) |
| H24A | 0.807309 | 1.070282 | 0.684668 | 0.042 |
| H24B | 0.864185 | 0.946401 | 0.766268 | 0.042 |
| H24C | 0.824291 | 1.104188 | 0.772367 | 0.042 |
| C25 | 0.45548(15) | 1.15344(13) | 0.70611(8) | 0.0199(2) |
| C26 | 0.28657(16) | 1.11684(14) | 0.70628(10) | 0.0274(3) |
| H26A | 0.219203 | 1.201190 | 0.676759 | 0.041 |
| H26B | 0.252974 | 1.083145 | 0.763977 | 0.041 |
| H26C | 0.279795 | 1.042837 | 0.678214 | 0.041 |
| C27 | 0.46492(17) | 1.27303(13) | 0.74761(9) | 0.0257(3) |
| H27A | 0.573123 | 1.294496 | 0.749080 | 0.039 |
| H27B | 0.427129 | 1.243328 | 0.804679 | 0.039 |
| H27C | 0.400507 | 1.357444 | 0.715511 | 0.039 |
| C28 | 0.50720(18) | 1.20561(15) | 0.61543(9) | 0.0285(3) |
| H28A | 0.508084 | 1.128760 | 0.589019 | 0.043 |
| H28B | 0.611892 | 1.236256 | 0.614744 | 0.043 |
| H28C | 0.434594 | 1.285106 | 0.584686 | 0.043 |

*U*_eq_ is defined as 1/3 of the trace of the orthogonalized *U_ij_* tensor.

**Table 1 Anisotropic displacement parameters [Å^2^] for Werz_ND165_a. The anisotropic displacement factor exponent takes the form: −2π^2^[ *h*^2^*(a*)*^2^*U*_11_ *+ k*^2^*(b*)*^2^*U*_22_ + … + *2hka*b*U*_12_ ]**

| **Atom** | ***U*_11_** | ***U*_22_** | ***U*_33_** | ***U*_23_** | ***U*_13_** | ***U*_12_** |
| --- | --- | --- | --- | --- | --- | --- |
| Si1 | 0.02651(18) | 0.01413(16) | 0.01906(17) | −0.00363(12) | 0.00531(13) | −0.00336(13) |
| Si2 | 0.02123(17) | 0.01243(15) | 0.01820(16) | −0.00158(12) | 0.00000(12) | −0.00212(12) |
| O1 | 0.0221(4) | 0.0145(4) | 0.0189(4) | −0.0015(3) | 0.0041(3) | −0.0038(3) |
| O2 | 0.0231(4) | 0.0111(4) | 0.0188(4) | −0.0008(3) | 0.0009(3) | −0.0018(3) |
| O3 | 0.0399(6) | 0.0228(5) | 0.0411(6) | 0.0017(4) | 0.0075(5) | 0.0087(4) |
| O4 | 0.0228(5) | 0.0310(5) | 0.0319(5) | −0.0038(4) | 0.0039(4) | −0.0021(4) |
| C1 | 0.0218(6) | 0.0138(5) | 0.0163(5) | −0.0029(4) | 0.0025(4) | −0.0050(4) |
| C2 | 0.0230(6) | 0.0131(5) | 0.0161(5) | −0.0032(4) | 0.0004(4) | −0.0026(4) |
| C3 | 0.0240(6) | 0.0160(6) | 0.0197(6) | −0.0033(4) | 0.0020(5) | −0.0027(5) |
| C4 | 0.0265(6) | 0.0157(6) | 0.0202(6) | −0.0049(5) | 0.0004(5) | −0.0004(5) |
| C5 | 0.0305(7) | 0.0134(5) | 0.0184(6) | −0.0012(4) | −0.0003(5) | −0.0015(5) |
| C6 | 0.0272(6) | 0.0158(6) | 0.0178(6) | −0.0027(4) | 0.0021(5) | −0.0043(5) |
| C7 | 0.0236(6) | 0.0141(5) | 0.0160(5) | −0.0039(4) | 0.0007(4) | −0.0033(4) |
| C8 | 0.0239(6) | 0.0146(5) | 0.0152(5) | −0.0039(4) | 0.0009(4) | −0.0043(4) |
| C9 | 0.0263(6) | 0.0162(5) | 0.0194(6) | −0.0021(4) | 0.0029(5) | −0.0053(5) |
| C10 | 0.0229(6) | 0.0202(6) | 0.0200(6) | −0.0029(5) | 0.0027(5) | −0.0059(5) |
| C11 | 0.0215(6) | 0.0200(6) | 0.0206(6) | −0.0035(5) | −0.0005(5) | −0.0035(5) |
| C12 | 0.0221(6) | 0.0153(5) | 0.0200(6) | −0.0020(4) | −0.0009(5) | −0.0031(4) |
| C13 | 0.0221(6) | 0.0155(5) | 0.0161(5) | −0.0037(4) | 0.0003(4) | −0.0041(4) |
| C14 | 0.0233(6) | 0.0116(5) | 0.0158(5) | −0.0013(4) | 0.0001(4) | −0.0028(4) |
| C15 | 0.0285(7) | 0.0207(6) | 0.0286(7) | −0.0043(5) | 0.0039(5) | 0.0001(5) |
| C16 | 0.0238(6) | 0.0216(6) | 0.0273(7) | −0.0016(5) | −0.0010(5) | −0.0022(5) |
| C17 | 0.0471(9) | 0.0264(7) | 0.0286(7) | −0.0123(6) | 0.0090(6) | −0.0136(6) |
| C18 | 0.0316(7) | 0.0262(7) | 0.0198(6) | −0.0031(5) | 0.0012(5) | 0.0007(5) |
| C19 | 0.0301(7) | 0.0171(6) | 0.0272(7) | −0.0011(5) | 0.0100(5) | −0.0004(5) |
| C20 | 0.0319(8) | 0.0285(8) | 0.0512(10) | 0.0069(7) | 0.0151(7) | 0.0069(6) |
| C21 | 0.0459(9) | 0.0296(7) | 0.0330(8) | −0.0100(6) | 0.0197(7) | −0.0048(6) |
| C22 | 0.0302(7) | 0.0287(7) | 0.0348(8) | −0.0050(6) | 0.0072(6) | −0.0085(6) |
| C23 | 0.0418(8) | 0.0201(6) | 0.0204(6) | −0.0031(5) | −0.0013(6) | −0.0074(6) |
| C24 | 0.0237(7) | 0.0211(6) | 0.0372(8) | −0.0042(5) | −0.0023(6) | −0.0032(5) |
| C25 | 0.0233(6) | 0.0147(5) | 0.0206(6) | −0.0036(4) | 0.0000(5) | −0.0006(4) |
| C26 | 0.0227(6) | 0.0206(6) | 0.0377(8) | −0.0067(5) | −0.0020(6) | 0.0004(5) |
| C27 | 0.0308(7) | 0.0160(6) | 0.0301(7) | −0.0069(5) | −0.0014(5) | 0.0002(5) |
| C28 | 0.0354(8) | 0.0232(6) | 0.0221(6) | 0.0002(5) | −0.0012(5) | 0.0021(5) |

**Table 1 Bond lengths and angles for Werz_ND165_a**

| **Atom–Atom** | **Length [Å]** |
| --- | --- |
| Si1–O1 | 1.6771(10) |
| Si1–C18 | 1.8566(14) |
| Si1–C17 | 1.8647(15) |
| Si1–C19 | 1.8863(15) |
| Si2–O2 | 1.6830(9) |
| Si2–C23 | 1.8573(14) |
| Si2–C24 | 1.8595(15) |
| Si2–C25 | 1.8905(13) |
| O1–C1 | 1.3774(14) |
| O2–C14 | 1.3747(14) |
| O3–C15 | 1.2049(17) |
| O4–C16 | 1.2087(17) |
| C1–C14 | 1.3618(17) |
| C1–C2 | 1.4410(16) |
| C2–C3 | 1.4109(18) |
| C2–C7 | 1.4158(17) |
| C3–C4 | 1.3801(17) |
| C3–H3 | 0.9500 |
| C4–C5 | 1.4076(18) |
| C4–C15 | 1.4762(19) |
| C5–C6 | 1.3698(18) |
| C5–H5 | 0.9500 |
| C6–C7 | 1.4183(16) |
| C6–H6 | 0.9500 |
| C7–C8 | 1.4497(17) |
| C8–C9 | 1.4190(17) |
| C8–C13 | 1.4200(16) |
| C9–C10 | 1.3718(18) |
| C9–H9 | 0.9500 |
| C10–C11 | 1.4078(17) |
| C10–H10 | 0.9500 |
| C11–C12 | 1.3804(17) |
| C11–C16 | 1.4769(19) |
| C12–C13 | 1.4077(18) |
| C12–H12 | 0.9500 |
| C13–C14 | 1.4443(17) |
| C15–H15 | 0.9500 |
| C16–H16 | 0.9500 |
| C17–H17A | 0.9800 |
| C17–H17B | 0.9800 |
| C17–H17C | 0.9800 |
| C18–H18A | 0.9800 |
| C18–H18B | 0.9800 |
| C18–H18C | 0.9800 |
| C19–C20 | 1.534(2) |
| C19–C22 | 1.535(2) |
| C19–C21 | 1.541(2) |
| C20–H20A | 0.9800 |
| C20–H20B | 0.9800 |
| C20–H20C | 0.9800 |
| C21–H21A | 0.9800 |
| C21–H21B | 0.9800 |
| C21–H21C | 0.9800 |
| C22–H22A | 0.9800 |
| C22–H22B | 0.9800 |
| C22–H22C | 0.9800 |
| C23–H23A | 0.9800 |
| C23–H23B | 0.9800 |
| C23–H23C | 0.9800 |
| C24–H24A | 0.9800 |
| C24–H24B | 0.9800 |
| C24–H24C | 0.9800 |
| C25–C26 | 1.5363(19) |
| C25–C28 | 1.5366(18) |
| C25–C27 | 1.5385(18) |
| C26–H26A | 0.9800 |
| C26–H26B | 0.9800 |
| C26–H26C | 0.9800 |
| C27–H27A | 0.9800 |
| C27–H27B | 0.9800 |
| C27–H27C | 0.9800 |
| C28–H28A | 0.9800 |
| C28–H28B | 0.9800 |
| C28–H28C | 0.9800 |
|  |  |
| **Atom–Atom–Atom** | **Angle [°]** |
| O1–Si1–C18 | 110.27(6) |
| O1–Si1–C17 | 108.82(6) |
| C18–Si1–C17 | 109.33(7) |
| O1–Si1–C19 | 105.51(6) |
| C18–Si1–C19 | 110.43(6) |
| C17–Si1–C19 | 112.41(7) |
| O2–Si2–C23 | 108.03(6) |
| O2–Si2–C24 | 110.38(6) |
| C23–Si2–C24 | 110.02(7) |
| O2–Si2–C25 | 106.17(5) |
| C23–Si2–C25 | 112.51(6) |
| C24–Si2–C25 | 109.65(6) |
| C1–O1–Si1 | 122.58(8) |
| C14–O2–Si2 | 124.32(8) |
| C14–C1–O1 | 121.86(10) |
| C14–C1–C2 | 120.50(11) |
| O1–C1–C2 | 117.61(11) |
| C3–C2–C7 | 118.98(11) |
| C3–C2–C1 | 120.31(11) |
| C7–C2–C1 | 120.71(11) |
| C4–C3–C2 | 121.06(12) |
| C4–C3–H3 | 119.5 |
| C2–C3–H3 | 119.5 |
| C3–C4–C5 | 120.21(12) |
| C3–C4–C15 | 118.16(12) |
| C5–C4–C15 | 121.64(11) |
| C6–C5–C4 | 119.47(11) |
| C6–C5–H5 | 120.3 |
| C4–C5–H5 | 120.3 |
| C5–C6–C7 | 121.78(12) |
| C5–C6–H6 | 119.1 |
| C7–C6–H6 | 119.1 |
| C2–C7–C6 | 118.49(11) |
| C2–C7–C8 | 118.91(11) |
| C6–C7–C8 | 122.60(11) |
| C9–C8–C13 | 118.62(12) |
| C9–C8–C7 | 122.52(11) |
| C13–C8–C7 | 118.85(11) |
| C10–C9–C8 | 121.51(12) |
| C10–C9–H9 | 119.2 |
| C8–C9–H9 | 119.2 |
| C9–C10–C11 | 119.63(12) |
| C9–C10–H10 | 120.2 |
| C11–C10–H10 | 120.2 |
| C12–C11–C10 | 120.13(12) |
| C12–C11–C16 | 118.35(12) |
| C10–C11–C16 | 121.49(12) |
| C11–C12–C13 | 121.25(11) |
| C11–C12–H12 | 119.4 |
| C13–C12–H12 | 119.4 |
| C12–C13–C8 | 118.84(11) |
| C12–C13–C14 | 120.48(11) |
| C8–C13–C14 | 120.68(11) |
| C1–C14–O2 | 121.67(11) |
| C1–C14–C13 | 120.11(11) |
| O2–C14–C13 | 118.15(11) |
| O3–C15–C4 | 125.65(13) |
| O3–C15–H15 | 117.2 |
| C4–C15–H15 | 117.2 |
| O4–C16–C11 | 125.39(12) |
| O4–C16–H16 | 117.3 |
| C11–C16–H16 | 117.3 |
| Si1–C17–H17A | 109.5 |
| Si1–C17–H17B | 109.5 |
| H17A–C17–H17B | 109.5 |
| Si1–C17–H17C | 109.5 |
| H17A–C17–H17C | 109.5 |
| H17B–C17–H17C | 109.5 |
| Si1–C18–H18A | 109.5 |
| Si1–C18–H18B | 109.5 |
| H18A–C18–H18B | 109.5 |
| Si1–C18–H18C | 109.5 |
| H18A–C18–H18C | 109.5 |
| H18B–C18–H18C | 109.5 |
| C20–C19–C22 | 108.19(13) |
| C20–C19–C21 | 108.99(12) |
| C22–C19–C21 | 109.23(11) |
| C20–C19–Si1 | 112.10(9) |
| C22–C19–Si1 | 109.88(9) |
| C21–C19–Si1 | 108.40(11) |
| C19–C20–H20A | 109.5 |
| C19–C20–H20B | 109.5 |
| H20A–C20–H20B | 109.5 |
| C19–C20–H20C | 109.5 |
| H20A–C20–H20C | 109.5 |
| H20B–C20–H20C | 109.5 |
| C19–C21–H21A | 109.5 |
| C19–C21–H21B | 109.5 |
| H21A–C21–H21B | 109.5 |
| C19–C21–H21C | 109.5 |
| H21A–C21–H21C | 109.5 |
| H21B–C21–H21C | 109.5 |
| C19–C22–H22A | 109.5 |
| C19–C22–H22B | 109.5 |
| H22A–C22–H22B | 109.5 |
| C19–C22–H22C | 109.5 |
| H22A–C22–H22C | 109.5 |
| H22B–C22–H22C | 109.5 |
| Si2–C23–H23A | 109.5 |
| Si2–C23–H23B | 109.5 |
| H23A–C23–H23B | 109.5 |
| Si2–C23–H23C | 109.5 |
| H23A–C23–H23C | 109.5 |
| H23B–C23–H23C | 109.5 |
| Si2–C24–H24A | 109.5 |
| Si2–C24–H24B | 109.5 |
| H24A–C24–H24B | 109.5 |
| Si2–C24–H24C | 109.5 |
| H24A–C24–H24C | 109.5 |
| H24B–C24–H24C | 109.5 |
| C26–C25–C28 | 109.06(11) |
| C26–C25–C27 | 109.40(11) |
| C28–C25–C27 | 108.35(11) |
| C26–C25–Si2 | 111.81(9) |
| C28–C25–Si2 | 111.16(9) |
| C27–C25–Si2 | 106.97(9) |
| C25–C26–H26A | 109.5 |
| C25–C26–H26B | 109.5 |
| H26A–C26–H26B | 109.5 |
| C25–C26–H26C | 109.5 |
| H26A–C26–H26C | 109.5 |
| H26B–C26–H26C | 109.5 |
| C25–C27–H27A | 109.5 |
| C25–C27–H27B | 109.5 |
| H27A–C27–H27B | 109.5 |
| C25–C27–H27C | 109.5 |
| H27A–C27–H27C | 109.5 |
| H27B–C27–H27C | 109.5 |
| C25–C28–H28A | 109.5 |
| C25–C28–H28B | 109.5 |
| H28A–C28–H28B | 109.5 |
| C25–C28–H28C | 109.5 |
| H28A–C28–H28C | 109.5 |
| H28B–C28–H28C | 109.5 |

**Table 1 Torsion angles for Werz_ND165_a**

| **Atom–Atom–Atom–Atom** | **Torsion Angle [°]** |
| --- | --- |
| C18–Si1–O1–C1 | 85.40(10) |
| C17–Si1–O1–C1 | −34.51(11) |
| C19–Si1–O1–C1 | −155.34(9) |
| C23–Si2–O2–C14 | −35.66(11) |
| C24–Si2–O2–C14 | 84.68(11) |
| C25–Si2–O2–C14 | −156.55(9) |
| Si1–O1–C1–C14 | −83.60(13) |
| Si1–O1–C1–C2 | 98.40(12) |
| C14–C1–C2–C3 | −174.60(12) |
| O1–C1–C2–C3 | 3.43(17) |
| C14–C1–C2–C7 | 4.50(18) |
| O1–C1–C2–C7 | −177.47(11) |
| C7–C2–C3–C4 | 1.10(18) |
| C1–C2–C3–C4 | −179.79(12) |
| C2–C3–C4–C5 | −0.44(19) |
| C2–C3–C4–C15 | 179.60(12) |
| C3–C4–C5–C6 | −0.57(19) |
| C15–C4–C5–C6 | 179.39(12) |
| C4–C5–C6–C7 | 0.92(19) |
| C3–C2–C7–C6 | −0.75(17) |
| C1–C2–C7–C6 | −179.86(11) |
| C3–C2–C7–C8 | 178.93(11) |
| C1–C2–C7–C8 | −0.18(17) |
| C5–C6–C7–C2 | −0.25(18) |
| C5–C6–C7–C8 | −179.91(12) |
| C2–C7–C8–C9 | 177.63(11) |
| C6–C7–C8–C9 | −2.70(19) |
| C2–C7–C8–C13 | −2.96(17) |
| C6–C7–C8–C13 | 176.71(11) |
| C13–C8–C9–C10 | −0.79(18) |
| C7–C8–C9–C10 | 178.61(12) |
| C8–C9–C10–C11 | −0.30(19) |
| C9–C10–C11–C12 | 0.97(19) |
| C9–C10–C11–C16 | −176.80(12) |
| C10–C11–C12–C13 | −0.53(19) |
| C16–C11–C12–C13 | 177.31(12) |
| C11–C12–C13–C8 | −0.57(18) |
| C11–C12–C13–C14 | 179.18(12) |
| C9–C8–C13–C12 | 1.22(17) |
| C7–C8–C13–C12 | −178.21(11) |
| C9–C8–C13–C14 | −178.53(11) |
| C7–C8–C13–C14 | 2.04(17) |
| O1–C1–C14–O2 | −0.36(18) |
| C2–C1–C14–O2 | 177.58(11) |
| O1–C1–C14–C13 | 176.59(11) |
| C2–C1–C14–C13 | −5.46(18) |
| Si2–O2–C14–C1 | −89.28(13) |
| Si2–O2–C14–C13 | 93.71(12) |
| C12–C13–C14–C1 | −177.52(11) |
| C8–C13–C14–C1 | 2.23(18) |
| C12–C13–C14–O2 | −0.46(17) |
| C8–C13–C14–O2 | 179.29(10) |
| C3–C4–C15–O3 | −173.84(14) |
| C5–C4–C15–O3 | 6.2(2) |
| C12–C11–C16–O4 | 175.21(13) |
| C10–C11–C16–O4 | −7.0(2) |
| O1–Si1–C19–C20 | 61.87(12) |
| C18–Si1–C19–C20 | −178.99(11) |
| C17–Si1–C19–C20 | −56.59(14) |
| O1–Si1–C19–C22 | −58.48(10) |
| C18–Si1–C19–C22 | 60.67(12) |
| C17–Si1–C19–C22 | −176.94(10) |
| O1–Si1–C19–C21 | −177.78(9) |
| C18–Si1–C19–C21 | −58.64(11) |
| C17–Si1–C19–C21 | 63.76(11) |
| O2–Si2–C25–C26 | 47.46(10) |
| C23–Si2–C25–C26 | −70.50(11) |
| C24–Si2–C25–C26 | 166.71(10) |
| O2–Si2–C25–C28 | −74.69(10) |
| C23–Si2–C25–C28 | 167.35(9) |
| C24–Si2–C25–C28 | 44.56(11) |
| O2–Si2–C25–C27 | 167.20(8) |
| C23–Si2–C25–C27 | 49.24(10) |
| C24–Si2–C25–C27 | −73.55(10) |

[1]	G. R. Fulmer, A. J. M. Miller, N. H. Sherden, H. E. Gottlieb, A. Nudelman, B. M. Stoltz, J. E. Bercaw, K. I. Goldberg, *Organometallics* **2010**, *29*, 2176.

[2]	J. Bitenc, K. Pirnat, E. Žagar, A. Randon-Vitanova, R. Dominko, *Journal of Power Sources* **2019**, *430*, 90.

[3]	A. Vizintin, J. Bitenc, A. Kopač Lautar, J. Grdadolnik, A. Randon Vitanova, K. Pirnat, *ChemSusChem* **2020**, *13*, 2328.

[4]	J. Bitenc, K. Pirnat, T. Bančič, M. Gaberšček, B. Genorio, A. Randon-Vitanova, R. Dominko, *ChemSusChem* **2015**, *8*, 4128.

[5]	H. Dong, O. Tutusaus, Y. Liang, Y. Zhang, Z. Lebens-Higgins, W. Yang, R. Mohtadi, Y. Yao, *Nat. Energy* **2020**, *5*, 1043.

[6]	Y. Ding, X. Ren, D. Chen, F. Wen, T. Li, F. Xu, *ACS Appl. Energy Mater.* **2022**, *5*, 3004.

[7]	X. Gao, X. Qi, X. Zhao, *Energy & Fuels* **2024**, *38*, 12221.

[8]	a) J. Wrobel, J. M. Cook, *Synth. Commun.* **1980**, *10*, 333; b) X. Guo, Y. Zhang, H. Chen, C. Cui, Z. Li, Y. Du, B. Wang, H. Yang, *International Journal of Electrochemical Science* **2020**, *15*, 7774.

[9]	T. Pavčnik, J. Imperl, M. Kolar, R. Dominko, J. Bitenc, *J. Mater. Chem. A* **2024**, *12*, 3386.

[10]	a) R. Ahlrichs, M. Bär, M. Häser, H. Horn, C. Kölmel, *Chem. Phys. Lett.* **1989**, *162*, 165; b) M. von Arnim, R. Ahlrichs, *J. Comput. Chem.* **1998**, *19*, 1746.

[11]	O. Treutler, R. Ahlrichs, *J. Chem. Phys.* **1995**, *102*, 346.

[12]	a) M. Sierka, A. Hogekamp, R. Ahlrichs, *J. Chem. Phys.* **2003**, *118*, 9136; b) R. Ahlrichs, *Phys. Chem. Chem. Phys.* **2004**, *6*, 5119.

[13]	F. Weigend, *Phys. Chem. Chem. Phys.* **2006**, *8*, 1057.

[14]	a) S. Grimme, J. Antony, S. Ehrlich, H. Krieg, *J. Chem. Phys.* **2010**, *132*, 154104; b) S. Grimme, S. Ehrlich, L. Goerigk, *J. Comput. Chem.* **2011**, *32*, 1456.

[15]	a) J. C. Slater, *Phys. Rev.* **1951**, *81*, 385; b) S. H. Vosko, L. Wilk, M. Nusair, *Can. J. Phys.* **1980**, *58*, 1200; c) A. D. Becke, *Phys. Rev. A* **1988**, *38*, 3098; d) John P. Perdew, *Phys. Rev. B* **1986**, *33*, 8822.

[16]	F. Weigend, R. Ahlrichs, *Phys. Chem. Chem. Phys.* **2005**, *7*, 3297.

[17]	D. Püschner, *Quantitative Rechenverfahren der Theoretischen Chemie*, Springer Fachmedien Wiesbaden, Wiesbaden, **2017**.

[18]	H.-D. W. W. Göpel, *Statistische Thermodynamik*, Spektrum Akademischer Verlag, Heidelberg, Berlin, **2000**.

[19]	a) G. S. A. Klamt, *J. Chem. Soc., Perkin Trans. 2* **1993**, 799; b) A. K. F. Eckert, *AIChE J.* **2002**, *48*, 369.

[20]	K. Izutsu, *Electrochemistry in nonaqueous solutions*, Wiley-VCH, Weinheim, **2002**.

[21]	C. J. E. Riedel, *Anorganische Chemie*, De Gruyter, Berlin, Boston, **2015**.

[22]	W. Göpel, H.-D. Wiemhöfer, *Statistische Thermodynamik*, Spektrum Akademischer Verlag, Heidelberg, Berlin, **2000**.

[23]	A. A. Isse, A. Gennaro, *The journal of physical chemistry. B* **2010**, *114*, 7894.

[24]	C. P. Kelly, C. J. Cramer, D. G. Truhlar, *The journal of physical chemistry. B* **2006**, *110*, 16066.

[25]	a) Bruker, *SAINT*. Madison, Wisconsin, USA, Bruker AXS Inc.; b) L. Krause, R. Herbst-Irmer, G. M. Sheldrick, D. Stalke, *Journal of applied crystallography* **2015**, *48*, 3.

[26]	a) G. M. Sheldrick, *Acta crystallographica. Section C, Structural chemistry* **2015**, *71*, 3; b) G. M. Sheldrick, *Acta crystallographica. Section A, Foundations and advances* **2015**, *71*, 3.

[27]	C. R. Groom, I. J. Bruno, M. P. Lightfoot, S. C. Ward, *Acta crystallographica Section B, Structural science, crystal engineering and materials* **2016**, *72*, 171.

[28]	D. Kratzert, "Daniel Kratzerts SC-XRD tools", can be found under https://dkratzert.de/finalcif.html.
